# A Y178C rhodopsin mutation causes aggregation and comparatively severe retinal degeneration

**DOI:** 10.1038/s41420-025-02311-4

**Published:** 2025-01-29

**Authors:** Sreelakshmi Vasudevan, Paul S.–H. Park

**Affiliations:** Department of Ophthalmology and Visual Sciences, Case Western Reserve University, Cleveland, OH USA

**Keywords:** Retina, Mechanisms of disease, Retinal diseases

## Abstract

Rhodopsin is the light-activated G protein-coupled receptor that initiates vision in photoreceptor cells of the retina. Numerous mutations in rhodopsin promote receptor misfolding and aggregation, causing autosomal dominant retinitis pigmentosa, a progressive retinal degenerative disease. The mechanism by which these mutations cause photoreceptor cell death, and the role aggregation plays in this process is still unclear. We recently demonstrated with the P23H and G188R rhodopsin mutants that the severity of aggregation observed in vitro is also reflected in vivo and impacts the rate of retinal degeneration. A Y178C rhodopsin mutant was investigated here to determine if this relationship applies broadly among mutations that cause misfolding and aggregation of the receptor. In vitro characterization indicated the Y178C rhodopsin mutant exhibits similar properties to the more severely aggregating G188R rhodopsin mutant, where the mutant is mislocalized to the endoplasmic reticulum in HEK293 cells and form aggregates that cannot be rescued by treatment with the retinoid 9-*cis* retinal. Despite these similarities in vitro, the Y178C rhodopsin mutant promoted a more severe retinal degeneration compared to the G188R mutant in vivo in mice. Aggregates of the Y178C rhodopsin mutant labeled by the dye PROTEOSTAT were morphologically similar to those formed by both the P23H and G188R rhodopsin mutants. There was, however, significantly greater photoreceptor cell death occurring independently of PROTEOSTAT-labeled aggregates in mice expressing the Y178C rhodopsin mutant compared to those expressing either the P23H or G188R rhodopsin mutants. Here, we demonstrate that PROTEOSTAT-labeled aggregates are not the sole cause of photoreceptor cell death promoted by the Y178C rhodopsin mutation in vivo, and there may be alternate aggregate forms contributing to cell death in these mice.

## Introduction

Rhodopsin is a G protein-coupled receptor (GPCR) residing in the outer segment of rod photoreceptor cells of the retina. This GPCR is activated by light to initiate phototransduction, a G protein-mediated signaling cascade and the first step of vision. The light receptor plays a central role in vision and proper function and expression is required to maintain the health of rod photoreceptor cells [1]. Dysfunction in rhodopsin is a leading cause of autosomal dominant retinitis pigmentosa (adRP), a progressive retinal degenerative disease currently without a cure [2, 3]. There are over 100 mutations in the rhodopsin gene that have been identified in patients with retinal disease, mostly retinitis pigmentosa [4]. Many of these mutations have been studied in vitro to determine the molecular defects caused by the mutation. A majority of mutations with known molecular defect cause misfolding of the receptor [4]. Rhodopsin is not unique and there are several mutations in other GPCRs known to cause misfolding, aggregation and disease [5–8]. Both the mechanism by which rhodopsin misfolding leads to rod photoreceptor cell death and whether all misfolding rhodopsin mutations cause retinal degeneration by a common mechanism is unclear.

The classification of misfolding rhodopsin mutations has traditionally been made based on in vitro biochemical and cell biology observations, and can be broadly classified as complete or partial misfolding mutations [9]. Complete misfolding mutants are retained in the endoplasmic reticulum (ER) and cannot be rescued by or reconstituted with retinoids such as 11-*cis* retinal or 9-*cis* retinal [10–18]. Partial misfolding mutants may traffic properly to the plasma membrane and/or can be rescued by or reconstituted with retinoids, at least partially. More detailed characterization of the aggregation properties of misfolding mutants has revealed that mutations can be subdivided even further beyond the broad classification as complete and partial misfolding mutations [19]. Although much has been learned about misfolding mutations of rhodopsin in vitro, the effect of the mutations in vivo and what in vitro aspects are preserved in vivo is less clear.

The P23H mutation in rhodopsin was the first identified mutation in rhodopsin known to cause adRP and is classified as a partial misfolding mutation [9, 20]. This mutation has been studied extensively both in vitro and in vivo, with several animal models studied harboring this mutant form of rhodopsin. Until recently, this was the only misfolding rhodopsin mutation examined in animal models. Two knockin mouse models for adRP were recently compared that express misfolding rhodopsin mutants from different classes, the mentioned partial misfolding P23H mutation and the complete misfolding G188R mutation [21]. These studies demonstrated that misfolding mutations cause the receptor to aggregate both in vitro and in vivo. The complete misfolding G188R mutation results in a more severe aggregation profile in vitro, which manifests in vivo as greater aggregation and faster retinal degeneration compared to that promoted by the P23H mutation. It is unclear if similar trends occur with other mutations from the same class.

**Fig. 1:**
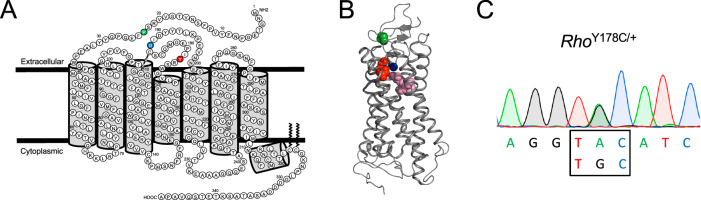
Y178C point mutation highlighted on the structure of rhodopsin. **A**, **B** Location of the P23H (green), G188R (blue), and Y178C (red) point mutations are highlighted on the murine rhodopsin secondary structure (**A**) and bovine rhodopsin crystal structure (**B**). In the crystal structure (PDB ID: 1U19), amino-acid side chains are shown as colored spheres, and 11-cis retinal is shown as pink spheres. **C** Sequence of codon 178 (box) and the surrounding region in rhodopsin transcripts from 2-week-old *Rho*^Y178C/+^ mice.

In the current study, we examined a Y178C mutation in rhodopsin under both in vitro and in vivo conditions to determine if it behaves similarly to either of the previously characterized P23H or G188R mutations. The mutation occurs in the second extracellular loop of rhodopsin and the native tyrosine residue is in close proximity to the bound ligand 11-*cis* retinal (Fig. 1A, B). The Y178C mutation in rhodopsin has been shown to cause adRP [22–25], and can cause a relatively severe phenotype [26]. This mutation can be classified as a complete misfolding mutation since it is retained in the ER and cannot bind retinoids [11, 13]. In this manner, it is predicted to behave more similarly to the G188R mutation than the P23H mutation.

## Results

### Rhodopsin Y178C mutant exhibits aggregation properties expected for a complete misfolding mutation

**Fig. 2:**
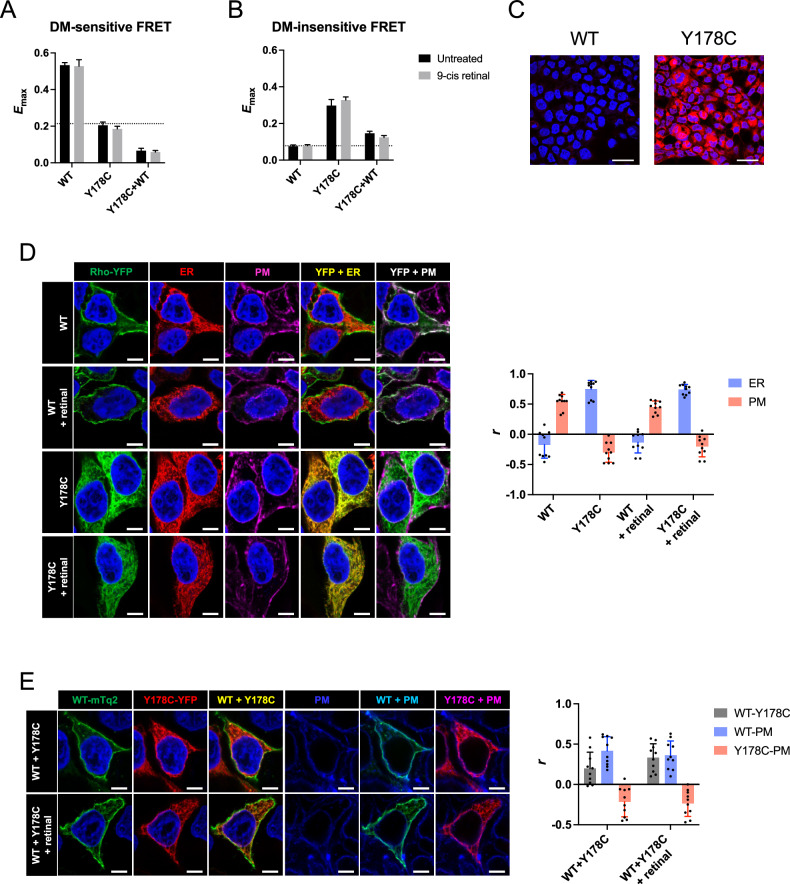
Aggregation and mislocalization of the Y178C rhodopsin mutant in HEK293 cells. **A**, **B** FRET was conducted on untreated (black) or 9-cis retinal-treated (gray) HEK239 cells either singly expressing or coexpressing WT and Y178C rhodopsin. Fitted values of the maximal FRET efficiency (*E*_max_) and the standard errors from the fits are shown for DM-sensitive (**A**) and DM-insensitive (**B**) components of generated FRET curves (Supplementary Fig. 1). The dashed line represents the non-specific *E*_max_, defined previously [19]. All fitted parameters and statistical analyses are reported in Supplementary Tables 1–3. **C** Transfected HEK239 cells expressing either untagged WT or Y178C rhodopsin were stained by PROTEOSTAT (red) and NucBlue (blue). Scale bar, 25 μm. **D** Transfected HEK293 cells expressing either YFP-tagged WT or Y178C rhodopsin were untreated or treated with 9-*cis* retinal. Overlays of confocal microscopy images of the YFP-tagged rhodopsins (green) with either the ER marker DsRed2-ER (red) or plasma membrane (PM) marker WGA (magenta) are shown on the left. DAPI staining is shown in blue. Scale bar, 5 μm. Colocalization analysis was conducted on the overlay confocal microscopy images to compute Pearson’s correlation coefficient (*r*), which indicates the presence or absence of colocalization between the YFP-tagged rhodopsins and the ER (blue) or PM (red) markers. Mean values and the standard deviation are shown (number of images, *n* = 10). **E** Transfected HEK293 cells coexpressing mTq2-tagged WT or YFP-tagged Y178C rhodopsin were untreated or treated with 9-*cis* retinal. Overlays of confocal images are shown on the left between mTq-tagged WT (green) and YFP-tagged Y178C (red) rhodopsin or between the tagged rhodopsins and the PM marker (blue). DAPI staining (blue) is shown only in images without the PM marker. Scale bar, 5 μm. The Pearson’s correlation coefficient (*r*) from colocalization analysis of overlay images is reported on the right as the mean and standard deviation (number of images, *n* = 10), which indicates the presence or absence of colocalization between WT and Y178C rhodopsin (gray), WT rhodopsin and PM marker (blue), or Y178C rhodopsin and PM marker (red).

Aggregation and mislocalization of the Y178C rhodopsin mutant were tested in vitro in transfected HEK293 cells to assess the molecular defect promoted by the mutation. The potential for the Y178C rhodopsin mutant to aggregate was tested using a Förster resonance energy transfer (FRET)-based method in transfected HEK293 cells [9]. The FRET originating from natively formed oligomers and non-native aggregates was differentiated by the sensitivity of the FRET signal to treatment with the mild detergent *n*-dodecyl-*β*-D-maltoside (DM), and only FRET above the non-specific FRET signal was considered an indication of physiologically relevant complexes [9]. Wild-type (WT) rhodopsin predominantly exhibited DM-sensitive FRET, indicative of oligomers, whereas the Y178C rhodopsin mutant predominantly exhibited DM-insensitive FRET, indicative of aggregates (Fig. 2A, B). As such, WT rhodopsin was predominantly properly localized to the plasma membrane whereas the Y178C rhodopsin mutant was predominantly mislocalized in the ER (Fig. 2D). To determine if the aggregates formed by the Y178C rhodopsin mutant can be detected by the dye PROTEOSTAT, a molecular rotor dye with greatly enhanced fluorescence upon binding aggregated proteins [27], HEK293 cells were transfected with constructs for untagged WT or Y178C rhodopsin since the fluorescent tags interfere with the fluorescence from the dye. Only cells expressing the Y178C rhodopsin mutant exhibited robust PROTEOSTAT staining (Fig. 2C), consistent with FRET data indicating only the Y178C rhodopsin mutant aggregates appreciably.

When WT and Y178 rhodopsin were coexpressed in HEK293 cells, specific FRET was largely absent except for a small DM-insensitive FRET signal (Fig. 2A, B), which originates from the interaction of a minor population of aggregated WT rhodopsin with the mutant, an interaction that may not occur physiologically in photoreceptor cells [28]. Confocal microscopy corroborated the FRET data, with WT rhodopsin localized in the plasma membrane, the Y178C mutant localized in the ER, and some colocalization of WT and Y178C rhodopsin (Fig. 2E). Thus, WT rhodopsin and the Y178C rhodopsin mutant are expected to behave independently of each other in vivo in photoreceptor cells. Treatment of cells with 9-*cis* retinal did not change any of the aggregation or localization profiles of the Y178C rhodopsin mutant when expressed alone or coexpressed with WT rhodopsin, thereby indicating that the Y178C mutant can be classified as a complete misfolding mutant.

### Rhodopsin Y178C mutant promotes a comparatively severe retinal degeneration phenotype

**Fig. 3:**
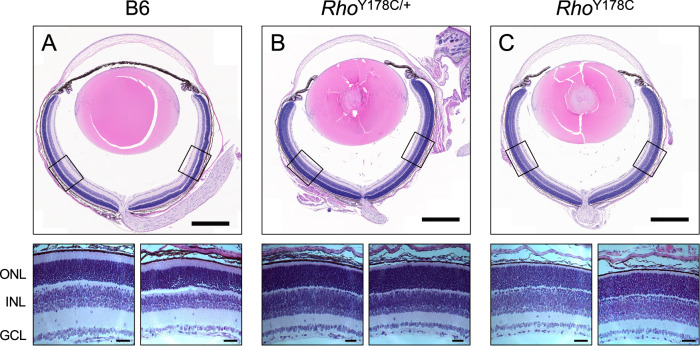
No loss of photoreceptor cells when rhodopsin is not expressed. Eye sections from 1-week-old B6 (**A**), *Rho*^Y178C/+^ (**B**), and *Rho*^Y178C^ (**C**) mice are shown with zoomed-in images shown below of regions of the retina highlighted by boxes. The outer nuclear layer (ONL), inner nuclear layer (INL), and ganglion cell layer (GCL) are labeled. Scale bar, 500 μm and 50 μm for zoomed-in images.

**Fig. 4:**
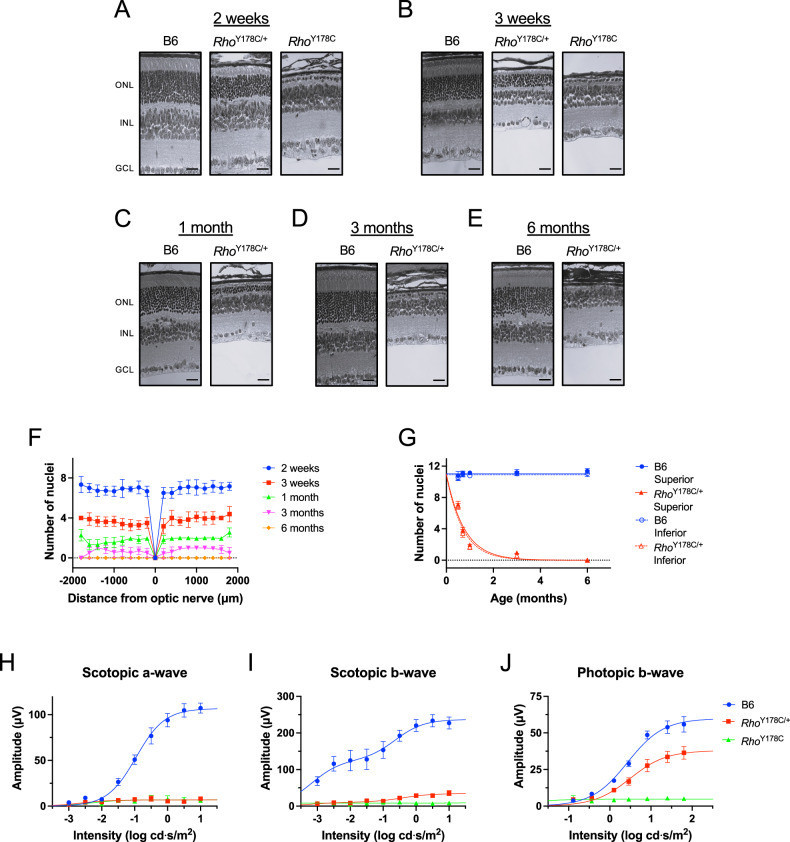
Photoreceptor cell loss in mice expressing the Y178C rhodopsin mutant. **A**–**E** Retinal sections from 2-week-old (**A**) and 3-week-old (**B**) B6, *Rho*^Y178C/+^ and *Rho*^Y178C^ mice and 1-month- (**C**), 3-month- (**D**), and 6-month-old (**E**) B6 and *Rho*^Y178C/+^ mice. The outer nuclear layer (ONL), inner nuclear layer (INL), and ganglion cell layer (GCL) are labeled. Scale bar, 25 μm. Uncropped images are shown in Supplementary Fig. 2. **F** Spider plot of the number of photoreceptor cell nuclei in the inferior (negative) and superior (positive) regions of the retina in *Rho*^Y178C/+^ mice ages 2 weeks–6 months. The mean and standard deviation are shown at different distances from the optic nerve (number of mice, *n* = 6 for all except for 6-month-old *Rho*^Y178C/+^ mice, where *n* = 2). Spider plots comparing B6, *Rho*^Y178C/+^ and *Rho*^Y178C^ mice at each age are shown in Supplementary Fig. 2. **G** Mean values of the number of photoreceptor cell nuclei, and their associated standard deviation, are plotted as a function of age in the superior or inferior region of the retina in B6 or *Rho*^Y178C/+^ mice (number of mice, *n* = 6 for all except for 6-month-old *Rho*^Y178C/+^ mice, where *n* = 2). Data points for B6 mice, besides that for 3-week-old mice, are those reported previously [21]. The kinetics of photoreceptor cell loss was determined by fitting the data by non-linear regression with an exponential equation for one-phase decay. The fitted values and standard error of the rate constant (*k*) for data from *Rho*^Y178C/+^ mice is 1.32 ± 0.06 month^−1^ and 1.42 ± 0.07 month^−1^, for the superior and inferior regions of the retina, respectively. **H**–**J** ERG response from 3-week-old B6, *Rho*^Y178C/+^ and *Rho*^Y178C^ mice. The amplitude of the scotopic a- (**H**) and b-wave (**I**) and photopic b-wave (**J**) are plotted as a function of the intensity of light stimulus. Mean values are plotted with the standard error (number of mice, *n* = 8 for B6 and *Rho*^Y178C^ mice and *n* = 9 for *Rho*^Y178C/+^ mice). Data were fit by non-linear regression to dose-response models, and fitted values are reported in Supplementary Table 4. Scotopic a-wave and b-wave data from *Rho*^Y178C/+^ and *Rho*^Y178C^ mice are shown on a magnified scale in Supplementary Fig. 3.

The phenotype promoted by the Y178C rhodopsin mutation was examined in mice that were heterozygous (*Rho*^Y178C/+^) or homozygous (*Rho*^Y178C^) for the mutation. These mice expressing the Y178C rhodopsin mutant were congenic on the C57Bl/6 J (B6) background [29]. Retinal degeneration was characterized by quantifying the number of photoreceptor cell nuclei spanning the outer nuclear layer. Photoreceptor cell loss was not evident in mice that were 1 week old (Fig. 3), an age where small amounts of rhodopsin are just beginning to be expressed [30]. Thus, photoreceptor cell loss requires rhodopsin expression. Photoreceptor cell loss was evident in young *Rho*^Y178C/+^ and *Rho*^Y178C^ mice at 2 weeks of age, a period where rhodopsin is beginning to be expressed at a rapidly increased rate [30], and it continued to progress with age (Fig. 4A–E). At 2 weeks of age, the loss of photoreceptor cells was less severe in *Rho*^Y178C/+^ mice, where 3–4 nuclei layers were lost, compared to *Rho*^Y178C^, where only a single row of nuclei remained (Fig. 4A). A complete loss of photoreceptor cells occurred by 6 months of age in *Rho*^Y178C/+^ mice (Fig. 4E). The loss of photoreceptor cells was similar in both the superior and inferior retina (Fig. 4F), with the rate of photoreceptor cell loss similar in both regions (Fig. 4G).

The functional consequence of the photoreceptor cell loss in 3-week-old *Rho*^Y178C/+^ and *Rho*^Y178C^ mice was examined by electroretinography (ERG). Both the scotopic and photopic response in *Rho*^Y178C^ mice was ablated (Fig. 4H–J), which is consistent with the severe retinal degeneration exhibited by these mice (Fig. 4B). In *Rho*^Y178C/+^ mice, where only about 1/3 of photoreceptor cells remained (Fig. 4B, F), the scotopic a-wave response was ablated and the scotopic b-wave response significantly diminished (Fig. 4H, I). A reduction in the photopic b-wave response also occurred in *Rho*^Y178C/+^ mice (Fig. 4J). The scotopic a-wave corresponds to the response from rod photoreceptor cells [31, 32], and thus the remaining 1/3 of photoreceptor cells in *Rho*^Y178C/+^ mice was unable to generate a detectable a-wave response. The scotopic b-wave corresponds to the response from bipolar cells, which receive input from all photoreceptor cells [33]. The scotopic b-wave response likely derives from the function of cone photoreceptor cells, which is also disrupted as revealed by the reduced photopic b-wave (Fig. 4J).

**Fig. 5:**
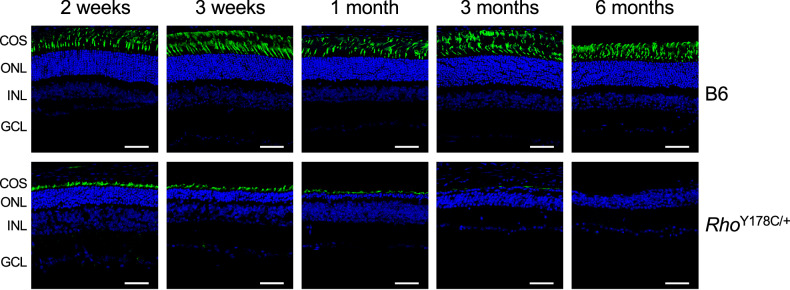
Outer segment shortening and loss of cone photoreceptor cells. Cone photoreceptor cells in the retina of 2-week-old–6-month-old B6 and *Rho*^Y178C/+^ mice were labeled by PNA (green), and nuclei were labeled by DAPI (blue). Cone outer segments (COS), outer nuclear layer (ONL), inner nuclear layer (INL), and ganglion cell layer (GCL) are labeled. Scale bar, 50 μm.

The reduced photopic b-wave suggests that cone photoreceptor cells are impacted in *Rho*^Y178C/+^ mice. Cone photoreceptor cells were visualized by peanut agglutinin (PNA) staining, which labels cone photoreceptor cell outer segments [34, 35]. Cone photoreceptor cell loss occurred progressively in *Rho*^Y178C/+^ mice, and complete cone photoreceptor cells loss occurred by 6 months of age (Fig. 5). The cone outer segments were also shorter in *Rho*^Y178C/+^ mice compared to B6 mice, even at 2 weeks of age.

### Rhodopsin expression in Rho^Y178C/+^ mice

**Fig. 6:**
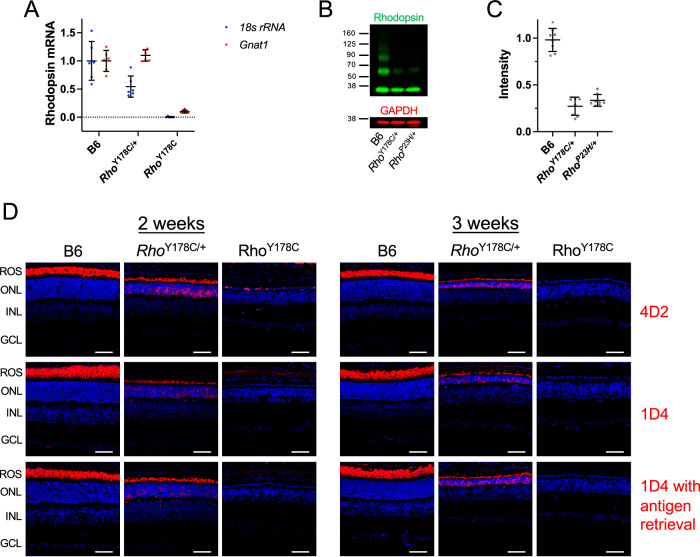
Expression and localization of rhodopsin in mice expressing the Y178C rhodopsin mutant. **A** Rhodopsin transcripts in the retina of 2-week-old B6, *Rho*^Y178C/+^ and *Rho*^Y178C^ mice were quantified by RT-qPCR and expressed normalized to *18* *s rRNA* (blue) or *Gnat1* (red). Individual data points are expressed relative to the mean value for B6 mice and are plotted along with the mean and standard deviation (number of mice, *n* = 6). Statistical analyses of data are in Supplementary Table 5. **B** Western blot of retinal extracts from 2-week-old B6 and *Rho*^Y178C/+^ mice and 1-month-old *Rho*^P23H/+^ mice. Blots were labeled with anti-1D4 (green) or anti-GAPDH (red) antibodies. **C** The level of rhodopsin in retinal extracts was determined by quantifying the intensity of bands in Western blots (e.g., Fig. 6B) and normalizing to the intensity of the band corresponding to GAPDH. Individual data points are expressed relative to the mean value for B6 mice and are plotted along with the mean and standard deviation (number of mice, *n* = 8). The difference between *Rho*^Y178C/+^ and *Rho*^P23H/+^ mice was not statistically significant (P > 0.05), as assessed by two-tailed t-test. **D** Retinal cryosections from 2-week- or 3-week-old B6, *Rho*^Y178C/+^ and *Rho*^Y178C^ mice were labeled with anti-4D2 or anti-1D4 antibodies (red). Anti-1D4 antibody labeling was performed both without and with an antigen retrieval step. Nuclei are labeled with DAPI (blue). Rod outer segments (ROS), outer nuclear layer (ONL), inner nuclear layer (INL), and ganglion cell layer (GCL) are labeled. Scale bar, 50 μm.

The expression of rhodopsin in 2-week-old *Rho*^Y178C/+^ and *Rho*^Y178C^ mice was examined at the transcript and protein levels. Transcripts of rhodopsin prepared from the retina of *Rho*^Y178C/+^ mice were sequenced to confirm that the only difference between the WT and mutant alleles was a single adenine to guanine base pair change corresponding to codon 178 in the rhodopsin sequence (Fig. 1C). RT-qPCR was conducted on retinal samples to quantify the level of rhodopsin transcripts, which were normalized to transcript levels of either 18 s rRNA or transducin (*Gnat1*) to account for the loss of photoreceptor cells (Fig. 6A). In *Rho*^Y178C/+^ mice, rhodopsin transcripts normalized to 18 s rRNA transcripts was about half of that detected in B6 mice. This decrease in rhodopsin transcripts was due to the loss of rod photoreceptor cells as rhodopsin transcripts were similar to that in B6 mice when normalized to transducin transcripts. Little or no rhodopsin transcripts were detected in *Rho*^Y178C^ mice, where almost all rod photoreceptor cells are lost.

Rhodopsin protein expression was assessed semi-quantitatively in Western blots (Fig. 6B). The level of rhodopsin in the retina of 2-week-old *Rho*^Y178C/+^ mice, as detected in Western blots, was about a quarter of that detected in B6 mice (Fig. 6C). Misfolded rhodopsin mutants appear to be predominantly degraded [36, 37], leaving mostly WT rhodopsin detected in Western blots of heterozygous mutant mice [21]. The level of rhodopsin detected in *Rho*^Y178C/+^ mice is only about half of the expected 50% level if WT rhodopsin expression is unaffected. Similar to the reduction in rhodopsin transcripts observed when normalized to 18 s rRNA transcripts (Fig. 6A), the reduction in rhodopsin protein likely is due to the loss of photoreceptor cells at this age tested. To support this conclusion, the level of rhodopsin expressed in the retina of *Rho*^P23H/+^ mice that were 1 month old, an age where comparable photoreceptor cell loss occurs [21], was determined by Western blot and showed a similar reduction in rhodopsin (Fig. 6C). Thus, the Y178C rhodopsin mutant, like the P23H and G188R rhodopsin mutants [21], appears to be efficiently degraded and most of the rhodopsin detected in Western blots appears to derive from the WT form.

The localization of rhodopsin in photoreceptor cells of 2-week- and 3-week-old *Rho*^Y178C/+^ and *Rho*^Y178C^ mice was examined by anti-4D2 and anti-1D4 antibodies, which detect the amino terminal region and carboxy terminus of rhodopsin, respectively [38, 39]. Previously in mice expressing either the P23H or G188R rhodopsin mutants, the anti-4D2 antibody was able to detect mislocalized rhodopsin whereas the anti-1D4 antibody was unable to detect its epitope in mislocalized rhodopsin without an antigen retrieval step, which was presumed to be an effect related to aggregation of the receptor [21]. Both antibodies labeled rhodopsin properly localized in the rod outer segment and mislocalized to the outer nuclear layer of the retina in *Rho*^Y178C/+^ mice at both ages (Fig. 6D). In 2-week-old *Rho*^Y178C^ mice, the anti-4D2 antibody labeled rhodopsin sporadically in the outer nuclear layer whereas the anti-1D4 antibody did not label any rhodopsin, even with antigen retrieval. Thus, residual levels of rod photoreceptor cells are present at this age and the anti-4D2 and anti-1D4 antibodies exhibit differential ability to detect the mutant rhodopsin in these photoreceptor cells. Neither antibody detected rhodopsin in 3-week-old *Rho*^Y178C^ mice, indicating the complete loss of rod photoreceptor cells.

Labeling of rhodopsin in the rod outer segment revealed that the length of this compartment is shorter in *Rho*^Y178C/+^ mice compared to that in B6 mice (Fig. 6D). Similar shortening of outer segments of cone photoreceptor cells (Fig. 5) indicates that the degeneration of both types of photoreceptor cells results in the shortening of the respective outer segments. The labeling of mislocalized rhodopsin in *Rho*^Y178C/+^ mice with the anti-1D4 antibody was similar regardless of whether or not an antigen retrieval step was included (Fig. 6D), which contrasts the labeling in the retina of *Rho*^P23H/+^ and *Rho*^G188R/+^ mice previously reported that required an antigen retrieval step for detection [21]. Thus, the structural nature of mislocalized rhodopsin in *Rho*^Y178C/+^ mice differs from that in *Rho*^P23H/+^ and *Rho*^G188R/+^ mice.

### Relationship between photoreceptor cell death and aggregation

**Fig. 7:**
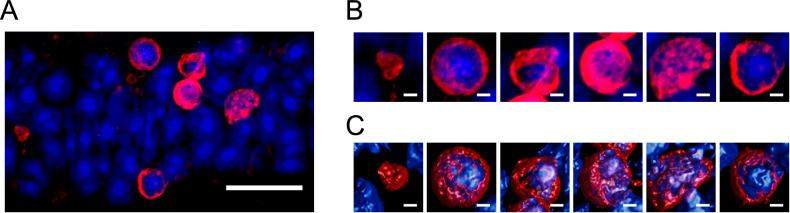
PROTEOSTAT labeling of aggregates in the retina of *Rho*^Y178C/+^ mice. **A** Retinal cryosections from 3-week-old *Rho*^Y178C/+^ mice were labeled with PROTEOSTAT (red) and NucBlue (blue). Scale bar, 10 μm. **B**, **C** Zoomed in images of individual nuclei with PROTEOSTAT labeling (**B**) and surface rendered images that show PROTEOSTAT staining surrounding the surface of the nuclei (**C**). Scale bar, 1 μm.

The Y178C rhodopsin mutant aggregates in vitro and can be labeled by PROTEOSTAT (Fig. 2C). The retina of *Rho*^Y178C/+^ mice were labeled with PROTEOSTAT to detect aggregates of the mutant rhodopsin in vivo. We previously demonstrated that aggregates of the P23H and G188R rhodopsin mutants can be detected by PROTEOSTAT in the retina of mice [21]. Characterization of the PROTEOSTAT staining in those mice demonstrated that neither retinal degeneration nor rhodopsin mislocalization by themselves causes aggregation of rhodopsin or any other protein that are detectable by PROTEOSTAT. Moreover, only PROTEOSTAT labeling in the outer nuclear layer of the retina was deemed to correspond to mutant rhodopsin aggregates as the origin of PROTEOSTAT labeling in other regions of the retina, including photoreceptor cell inner/outer segments, was ambiguous and may represent non-specific labeling since it also occurred in B6 mice. In the outer nuclear layer of the retina in *Rho*^Y178C/+^ mice, PROTEOSTAT labeling surrounded the nuclei of photoreceptor cells (Fig. 7). Perinuclear PROTEOSTAT labeling occurred for both relatively healthy nuclei with a single large central chromocenter as well as unhealthy disrupted nuclei. This morphology of PROTEOSTAT labeling was similar to that observed previously in the retina of mice expressing the P23H and G188R rhodopsin mutants [21].

**Fig. 8:**
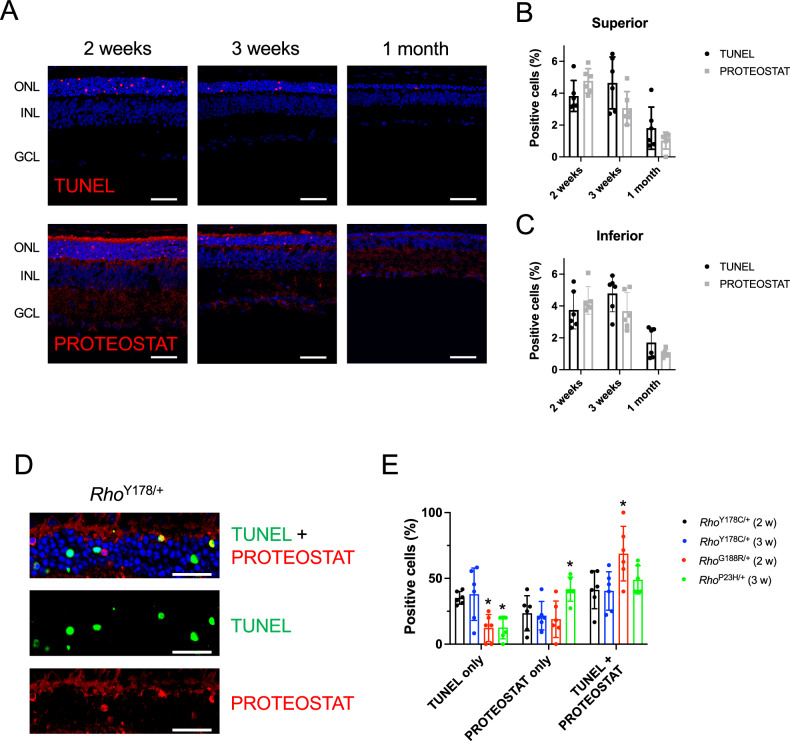
Relationship between PROTEOSTAT-labeled aggregates and photoreceptor cell death. **A** Retinal cryosections from 2-week-old–1-month-old *Rho*^Y178C/+^ mice were labeled by TUNEL or PROTEOSTAT (red). Nuclei were labeled by DAPI (blue). Scale bar, 50 μm. **B**, **C** The number of cells labeled by TUNEL or PROTEOSTAT in the outer nuclear layer were quantified in the superior (**B**) and inferior (**C**) regions of the retina. Individual data points are plotted along with the mean and standard deviation (number of mice, *n* = 6). Statistical analyses are reported in Supplementary Table 6. **D** Retinal cryosection from 3-week-old *Rho*^Y178C/+^ mice was labeled with both TUNEL (green) and PROTEOSTAT (red). Nuclei were labeled by NucBlue (blue). Scale bar, 25 μm. Retinal cryosections from 2-week-old *Rho*^Y178C/+^ mice, 2-week-old *Rho*^G188R/+^ mice, and 3-week-old *Rho*^P23H/+^ mice are shown in Supplementary Fig. 5. **E** The number of cells labeled by TUNEL or PROTEOSTAT alone or co-labeled with TUNEL and PROTEOSTAT were quantified from confocal microscopy images of retinal cryosections of 2-week-old *Rho*^Y178C/+^ and *Rho*^G188R/+^ mice and 3-week-old *Rho*^Y178C/+^ and *Rho*^P23H/+^ mice (e.g., Fig. 8D and Supplementary Fig. 5). Individual data points are plotted along with the mean and standard deviation (number of mice, *n* = 6). Data from *Rho*^G188R/+^ and *Rho*^P23H/+^ mice that are significantly different (*P* < 0.05) from *Rho*^Y178C/+^ mice are indicated by asterisks (*). Statistical analyses are reported in Supplementary Table 7.

The relationship between photoreceptor cell death and aggregation was assessed by examining the levels of TUNEL and PROTEOTAT positive photoreceptor cells, respectively (Fig. 8A–C). No TUNEL or PROTEOSTAT labeling was detected in the outer nuclear layer of B6 mice (Supplementary Fig. 4). The level of TUNEL positive cells was elevated at 2 weeks and 3 weeks of age in *Rho*^Y178C/+^ mice, and then decreased by 1 month of age. In contrast, the peak detection of PROTEOSTAT positive cells occurred at 2 weeks of age and then decreased with age. The relationship between TUNEL and PROTEOSTAT positive photoreceptor cells was further examined by determining how many photoreceptor cells were co-labeled (Fig. 8D). For both 2-week- and 3-week-old *Rho*^Y178C/+^ mice, a little under half the cells were co-labeled with TUNEL and PROTEOSTAT, whereas a little over half the cells were labeled with either TUNEL or PROTEOSTAT alone (Fig. 8E). *Rho*^P23H/+^ and *Rho*^G188R/+^ mice exhibited differences in the labeling pattern of TUNEL and PROTEOSTAT. The ages of these mice examined represented the age where peak levels of TUNEL and PROTEOSTAT positive cells occurred [21]. Both mutant mice exhibited about 3-fold lower levels of TUNEL only positive cells compared to that in *Rho*^Y178C/+^ mice. Thus, there was a greater proportion of photoreceptor cells exhibiting PROTEOSTAT only labeling or co-labeling with TUNEL and PROTEOSTAT in *Rho*^P23H/+^ and *Rho*^G188R/+^ mice compared to that in *Rho*^Y178C/+^ mice. The level of photoreceptor cells labeled with PROTEOSTAT alone was the greatest in *Rho*^P23H/+^ mice and *Rho*^G188R/+^ mice had the greatest level of photoreceptor cells co-labeled with TUNEL and PROTEOSTAT. Taken together, the relationship between photoreceptor cell death and aggregation appears to be less tightly linked in *Rho*^Y178C/+^ mice compared to that in *Rho*^P23H/+^ and *Rho*^G188R/+^ mice, and there are more photoreceptor cells undergoing photoreceptor cell death independent of aggregation. The aggregation detected here is limited to aggregate species labeled by PROTEOSTAT.

## Discussion

The Y178C rhodopsin mutation was examined both in vitro and in vivo to determine the molecular and cellular effects of the mutation. The classification as a complete misfolding mutation predicted that this mutation would behave similarly as a G188R rhodopsin mutation, which is another complete misfolding mutation characterized recently both in vitro and in vivo [19, 21, 28]. In vitro studies here indeed demonstrated that the Y178C rhodopsin mutant behaves similarly as the G188R rhodopsin mutant (Fig. 2). Both mutants are retained in the ER and form aggregates rather than oligomers. When coexpressed with WT rhodopsin, both mutants minimally interact with WT rhodopsin. The retinoid 9-*cis* retinal does not impact any of these properties for both mutants. This lack of effect of 9-*cis* retinal contrasts with the effect of the retinoid in the P23H rhodopsin mutant [19, 21, 40], which is classified as a partial misfolding mutant.

Previous characterizations of adRP mouse models expressing either the G188R or P23H rhodopsin mutants revealed that both mutants aggregated, and degeneration was faster in the inferior retina compared to the superior retina [21]. Mice expressing the G188R rhodopsin mutant exhibited a 2-fold faster loss of photoreceptor cells (Supplementary Fig. 6) and greater levels of photoreceptor cells labeled by PROTEOSTAT compared to mice expressing the P23H rhodopsin mutant [21]. Aggregation of these mutants appeared to be a primary driver of photoreceptor cell loss with the more severe aggregation profile of the G188R rhodopsin mutant observed both in vitro and in vivo correlating with a more severe retinal degeneration. Although in vitro studies indicate that the Y178C rhodopsin mutant shares a similar aggregation profile as the G188R rhodopsin mutant, the mouse model expressing the Y178C rhodopsin mutant exhibited key differences compared to the previously characterized mouse model of adRP that expresses the G188R rhodopsin mutant [21].

In *Rho*^Y178C/+^ mice, photoreceptor cell loss was 3-fold faster in the superior retina and 2-fold faster in the inferior retina compared to that in *Rho*^G188R/+^ mice (Supplementary Fig. 6) [21], and there was no difference in the degeneration occurring in the superior retina versus inferior retina (Fig. 4F, G). The relatively severe retinal degeneration phenotype in *Rho*^Y178C/+^ mice is consistent with the relatively severe phenotype observed in patients with adRP [26]. A difference in the time course of photoreceptor cell death, as assessed by TUNEL, is also observed between *Rho*^Y178C/+^ and *Rho*^G188R/+^ mice. While both mutant mice exhibit comparable levels of TUNEL positive cells early on at 2 weeks of age, levels decreased at 3 weeks of age in *Rho*^G188R/+^ mice whereas they remain elevated in *Rho*^Y178C/+^ mice (Fig. 8B, C). For both *Rho*^G188R/+^ and *Rho*^P23H/+^ mice, where aggregation appears to be a major driver of cell death, the level of TUNEL positive cells decreases relatively quickly 1 week after peak levels are achieved [21]. Thus, elevated levels of cell death are comparatively prolonged in *Rho*^Y178C/+^ mice.

In the absence of detectable differences in in vitro characterizations, what then could be the reason for the faster degeneration in *Rho*^Y178C/+^ mice compared to that in either *Rho*^G188R/+^ or *Rho*^P23H/+^ mice? Retinal degeneration required rhodopsin expression since photoreceptor cell loss is not evident at an age where rhodopsin expression is minimal (Fig. 3). The expression of rhodopsin transcripts and protein in *Rho*^Y178C/+^ mice are comparable to that in *Rho*^G188R/+^ and *Rho*^P23H/+^ mice (Fig. 6A–C) [21], and therefore this is not a factor in the observed difference. The morphology of PROTEOSTAT labeling that surround photoreceptor cell nuclei is qualitatively similar in *Rho*^Y178C/+^ mice compared to that in *Rho*^G188R/+^ and *Rho*^P23H/+^ mice (Fig. 7) [21], suggesting the Y178C rhodopsin mutant can form similar types of aggregates as that formed by the other two mutants, at least for those forming in the outer nuclear layer. The comparatively faster retinal degeneration in *Rho*^Y178C/+^ mice may be due to either an alternative type of aggregate formed that is not detectable by PROTEOSTAT or some other yet-to-be-determined factor. For instance, aggregates of the mutant may form and accumulate in the inner segment of rod photoreceptor cells, as it has been suggested to occur for a fluorescent protein-tagged P23H rhodopsin mutant in knockin mice [41]. Due to the ambiguity of PROTEOSTAT labeling outside of the outer nuclear layer [21], such an aggregate species may exist in mutant mice studied here and contribute to photoreceptor cell death but may not be distinguishable by PROTEOSTAT.

*Rho*^Y178C/+^ mice exhibit differences in the relationship between photoreceptor cell death and aggregation compared those in *Rho*^G188R/+^ and *Rho*^P23H/+^ mice. There was significantly more photoreceptor cell death occurring independent of aggregates, at least those labeled by PROTEOSTAT, in *Rho*^Y178C/+^ mice compared to that in the other mutant mice (Fig. 8E). Although PROTEOSTAT-labeled aggregates of the Y178C rhodopsin mutant appear to be a major contributor to photoreceptor cell death, since almost half of photoreceptor cells exhibit co-labeling by TUNEL and PROTEOSTAT (Fig. 8E), there is a comparatively large fraction of photoreceptor cells that die independently of PROTEOSTAT-labeled aggregates in the outer nuclear layer. In contrast, PROTEOSTAT-labeled aggregates in the outer nuclear layer appear to be the main driver of photoreceptor cell death in *Rho*^G188R/+^ and *Rho*^P23H/+^ mice [21].

The nature of the additional contributor to cell death besides PROTEOSTAT-labeled aggregates in *Rho*^Y178C/+^ mice is unclear, but immunohistochemistry with the anti-1D4 antibody suggests that perhaps there is an additional aggregate or misfolded species that is undetectable by PROTEOSTAT. Previously, it was assumed that the epitope detected by the anti-1D4 antibody is masked by rhodopsin mutant aggregation [21]. Here, the anti-1D4 antibody detected mislocalized rhodopsin in *Rho*^Y178C/+^ mice even without antigen retrieval (Fig. 6D), which indicates the presence of an alternate rhodopsin mutant species. In vitro experiments predict that Y178C and WT rhodopsin would not interact with each other appreciably in vivo and the comparable level of rhodopsin detected in Western blots from *Rho*^Y178C/+^ and *Rho*^P23H/+^ mice suggest that this mislocalized species is unrelated to WT rhodopsin (Figs. 2A, B and 6C). It is also notable that in 2-week-old *Rho*^Y178C^ mice, the anti-4D2 antibody exhibited some staining whereas the anti-1D4 antibody did not exhibit any staining, even with antigen retrieval (Fig. 6D), which was also observed in *Rho*^P23H^ mice at an age with comparable degeneration [21]. The nature of the mutant rhodopsin species selectively stained is unclear but may be yet another aggregate species or a proteolytically cleaved species (e.g., [42]).

The immunohistochemistry data described above point to the possibility that the Y178C rhodopsin mutant can exist in multiple forms, including possibly different types of aggregate species. Amyloid-type aggregates that cause neurodegeneration have been shown to adopt different forms and conformations, which can lead to heterogenous phenotypes [43]. Thus, it would not be surprising that different mutations in rhodopsin can likewise alter the forms or conformations achieved during aggregation, impacting the retinal degeneration phenotype. It is unclear whether the Y178C rhodopsin mutant forms a different aggregate species than the G188R rhodopsin mutant in vitro that are indistinguishable in our FRET assay. Moreover, it is also unclear whether mutant rhodopsin aggregates labeled by PROTEOSAT both in vitro and in vivo form the same type of aggregate species. Future efforts will require more structural information on rhodopsin mutant aggregation to better understand the different types of aggregate species formed by the receptor and the mechanism by which aggregation leads to photoreceptor cell death. The aggregation properties of different rhodopsin mutations that cause adRP can be variable in vitro [19], and the impact for those variations are only beginning to be understood in vivo (e.g., [21]). Here we demonstrate that even when mutations share similar in vitro aggregation properties, the effect of the mutation in vivo may be variable. Thus, more work is required to understand the correlation between in vitro and in vivo properties and the individual effect of mutations in disease.

## Materials and methods

### Mice

All animal studies were approved by the Institutional Animal Care and Use Committee at Case Western Reserve University School of Medicine. Both male and female mice were used for experiments. No randomization procedures or blinding were performed in animal studies. We previously generated *Rho*^G188R^ mice by CRISPR/Cas gene targeting [21]. *Rho*^Y178C/+^ (stock no. 043591-JAX) and *Rho*^P23H^ (stock no. 017628) mice were obtained from The Jackson Laboratory (Bar Harbor, ME). Both mouse lines were backcrossed with B6 mice for at least 10 generations. *Rho*^Y178C^ mice were generated by chemically induced ENU mutagenesis [29]. Absence of the rd1 and rd8 mutations and presence of the L450M mutation in RPE65 characteristic of B6 mice was confirmed by genotyping [44, 45]. A 10,000 base pair region of the genome containing the rhodopsin gene and promoter region was sequenced by PCR-amplifying overlapping fragments to confirm that mice exhibited no changes except for the TAC to TGC mutation at codon 178. To ensure proper generation of rhodopsin transcripts, total RNA was prepared from the retina of 2-week-old *Rho*^Y178C/+^ mice using High Pure RNA Tissue Kit (Roche Diagnostics, Indianapolis, IN) and reverse transcription performed using the Transcriptor First Strand cDNA Synthesis Kit (Roche Diagnostics, Indianapolis, IN). The cDNA was PCR-amplified using rhodopsin-specific primers (forward, 5’-CCTTGGTCTCTGTCTACGAAGAG; reverse, 5’–GAGCCTGCATGACCTCATC) and sequenced to confirm absence of changes except the TAC to TGC mutation at codon 178 (Fig. 1C).

### Characterization of aggregation in vitro in HEK293 cells

DNA constructs coding for murine rhodopsin in untagged form (pmRho) or tagged with a yellow fluorescent protein (YFP) variant or mTurquoise2 (mTq2), both tagged with a 1D4 epitope (pmRho-SYFP2-1D4 or pmRho-mTq2-1D4), were described previously [21, 46]. The Y178C mutation was introduced into each of these constructs adapting procedures in the QuickChange II Site-Directed Mutagenesis Kit (Agilent Technologies, Santa Clara, CA) using the following forward and reverse primers: 5’-GGCTGGTCCAGGTGCATCCCTGAGGGC and 5’-GCCCTCAGGGATGCACCTGGACCAGCC. These DNA constructs were used to transfect HEK293T/17 cells (Cat. No. CRL-11268, American Type Culture Collection, Manassas, VA), and a FRET assay or confocal microscopy performed as described previously [9, 46]. Cells were either untreated or treated with 15 μM 9-*cis* retinal (Cayman Chemical, Ann Arbor, MI) 3 h after transfection under dim red-light conditions and incubated in the dark.

The FRET assay was conducted on a FluoroMax-4 spectrofluorometer (Horiba Jobin Yvon, Edison, NJ), as described previously [9]. Total, *n*-dodecyl-*β*-D-maltoside (DM)-sensitive, and DM-insensitive FRET signals were computed and FRET curves generated by plotting the FRET efficiency (*E*) versus the acceptor:donor (A:D) ratio. The data were fit by non-linear regression to a rectangular hyperbolic function using Prism 10 (GraphPad Software, San Diego, CA), *E* = (*E*_max_ × A:D)/(EC_50_ + A:D), to compute the maximal FRET efficiency (*E*_max_) and EC_50_ [9]. The non-specific *E*_max_ was defined previously [19]. An extra sum of squares F test was conducted using Prism 10 (GraphPad Software, San Diego, CA) to determine if the FRET signal was different from the non-specific FRET signal and to determine differences among *E*_max_ values.

Confocal microscopy on transfected cells was performed on an SP8 confocal microscope (Leica, Buffalo Grove, IL) equipped with a 100×/1.4-NA oil objective, as described previously [19]. Cells were prepared for confocal microscopy, as described [46], and were labeled with DAPI (Bio-Rad, Hercules, CA) and wheat germ agglutinin (WGA)-Alexa Fluor 647 conjugate (Invitrogen, Carlsbad, CA) to label the nuclei and plasma membrane, respectively, and the ER was labeled by cotransfecting cells with pDsRed2-ER (Takara Bio USA, Mountain View, CA). Colocalization analysis was conducted in the Coloc 2 plugin in Fiji (version 2.1.0/1.53c) [47] to compute the Pearson’s correlation coefficient (*r*), as described previously [40].

Aggregates in HEK293T/17 cells were also detected with the PROTEOSTAT Aggresome Detection Kit (Enzo Life Sciences, Farmingdale, NY). Cells grown on poly-L-lysine treated #1.5 coverslip glass (Thermo Fisher Scientific, Waltham, MA) were transfected with DNA constructs coding for untagged WT or Y178C rhodopsin, as described previously [46]. 24 h post-transfection, cells were fixed, permeabilized and labeled with PROTEOSTAT, following the manufacturer’s protocol. Cells were mounted with ProLong Glass Antifade Mountant with NucBlue stain (Invitrogen, Carlsbad, CA) and imaged by confocal microscopy on an Olympus FV1200 IX83 laser scanning confocal microscope (Evident Scientific, Waltham, MA) using a UPLXAPO 100×/1.45 NA objective. PROTEOSTAT was detected by 559 nm diode laser excitation and 575–620 nm emission and NucBlue detected by 405 nm diode laser excitation and 425–460 nm emission.

### Characterization of retinal degeneration

Retinal degeneration was characterized histologically by analyzing hematoxylin and eosin (H&E)-stained retinal sections and functionally by electroretinography (ERG). For histology, retinal sections were prepared from mouse eyes, imaged on an Axio Scan.Z1 Slide Scanner equipped with a Hitachi HV-F203 camera and a Plan Apo 20×/0.8-NA objective (Carl Zeiss Microscopy, White Plains, NY) or a Leica DME compound microscope equipped with an EC3 digital camera and 40×/0.65-NA objective (Leica Microsystems, Buffalo, NY), and analyzed as described previously [21, 48]. The number of nuclei spanning the outer nuclear layer was counted manually in three different sections from the same eye and averaged. Kinetics of photoreceptor cell loss were determined by fitting averaged data from 600, 800, and 1000 μm from the optic nerve to an equation for one-phase decay ($y={(y}_{0}-{plateau})\times {e}^{-{kx}}+{plateau})$ using non-linear regression to obtain the rate constant (*k*) using Prism 10 (GraphPad Software, San Diego, CA). The parameter *y*_0_ was set to be common among data from either the superior or inferior retina and the parameter *plateau* was fixed at 0.

ERG was performed on dark-adapted mice under dim red-light conditions and data analyzed, as described previously [21]. ERG traces were collected on a Celeris rodent ERG system running Espion 6.0 software (Diagnosys, Lowell, MA) equipped with standard full-field stimulators with Ag/AgCl electrodes using touch/touch protocol. The a-wave and b-wave amplitudes from ERG traces obtained under scotopic conditions or the b-wave amplitudes from photopic responses obtained after 7 min of light adaptation at 20 cd·s/m^2^ were plotted and fit by non-linear regression in Prism 10 (GraphPad Software, San Diego, CA) to a standard dose-response model, $R=\frac{{R}_{\max }}{1+{10}^{\log {K}_{{\rm{A}}}-\log I}}$, or biphasic dose-response model, $R=\frac{{R}_{\max }\,\times {f}}{1+{10}^{\log {K}_{{\rm{A}}}-\log I}}+\,\frac{{R}_{\max }\,\times \,(1-f)}{1+{10}^{\log {K}_{{\rm{B}}}-\log I}}\,$. *R* is the amplitude of the a-wave or b-wave at a given flash intensity (*I*), *R*_max_ is the maximal amplitude at a saturating flash intensity, *K*_A_ and *K*_B_ represents the flash intensity that generates a half-maximal amplitude, *f* is the fraction of the curve that has *K*_A_.

### Characterization of rhodopsin expression

Transcripts of rhodopsin in the retina of mice were quantified by RT-qPCR conducted on the LightCycler 96 Real-Time PCR System (Roche Diagnostics, Indianapolis, IN), as described previously [49]. Primers for rhodopsin, transducin, and 18 s rRNA, were those described previously [21], and levels of rhodopsin transcripts were normalized to those of either transducin or 18 s rRNA. Rhodopsin protein levels in the retina of mice were quantified by Western blot analysis. Preparation of retina samples, SDS-PAGE using Novex 4–12% Tris-glycine gels (Invitrogen, Camarillo, CA), Western blotting procedures, and quantification of bands on Western blots by the Odyssey Fc Imaging System (LI-COR Biosciences, Lincoln, NE) were performed, as described previously [21]. Western blots were prepared using primary antibodies against rhodopsin (anti-1D4) [39] and GAPDH (Cat. No. 10494-1-AP; Proteintech, Rosemont, IL) and IRDye 800CW donkey anti-mouse (Cat. No. 926-32212) or IRDye 680LT donkey anti-rabbit (Cat. No. 925-68023) secondary antibodies (LI-COR Biosciences, Lincoln, NE).

### Confocal microscopy of labeled retinal cryosections

Retinal cryosection preparation and immunohistochemistry was conducted essentially as described previously [21, 50], using anti-1D4 [39] or anti-4D2 (Cat. No. MABN15, MilliporeSigma, Burlington, MA) primary antibodies and Alexa Flour 647 goat anti-mouse secondary antibody (Cat. No. A21237, Thermo Fisher Scientific, Waltham, MA). Retinal cryosections were incubated for 1 h in PBS containing 0.05% Tween 20 (USB, Cleveland, OH) supplemented with 1% goat serum (Invitrogen, Camarillo, CA) prior to incubation with antibodies. Antigen retrieval was achieved by incubating retinal cryosections in 10 mM Tris-HCl (pH 9) at 60 °C for 10 min and then at room temperature for 30 min. Cone photoreceptor cells were labeled with biotinylated PNA (Cat. No. B-1075, Vector Laboratories, Newark, CA) followed by streptavidin Alexa Fluor 488 conjugate (Cat. No. S11223, Thermo Fisher Scientific, Waltham, MA). TUNEL assay was conducted on retinal cryosections using DeadEnd Fluorometric TUNEL System (Promega, Madison, WI) and PROTEOSTAT labeling of retinal cryosections was conducted using the PROTEOSTAT Aggresome Detection Kit (Enzo Life Sciences, Farmingdale, NY), as described previously [21, 50]. When TUNEL assay and PROTEOSTAT co-labeling was performed on the same retinal cryosection, One-step TUNEL In Situ Apoptosis Kit (Elabscience, Houston, Tx) was used, according to the manufacturer’s instructions. Labeled cryosections were cover-slipped with DAPI Fluoromount-G mounting media (Southern Biotech, Birmingham, AL) for 40× imaging or with ProLong Glass Antifade Mountant with NucBlue stain (Invitrogen, Carlsbad, CA) for 100× imaging.

Labeled retinal cryosections were imaged by confocal microscopy on an Olympus FV1200 IX83 laser scanning confocal microscope (Evident Scientific, Waltham, MA) using either a UPlanXApo 40×/1.40 NA oil objective or UPLXAPO100×/1.45 NA objective, as described previously [21]. DAPI and NucBlue were detected with 405 nm diode laser excitation and 425–460 nm emission, CF 647 was detected with 635 nm diode laser excitation and 655–755 nm emission, TUNEL positive cells were detected by 473 nm argon-ion laser excitation and 485–542 nm emission or by 635 nm diode laser excitation and 655–755 nm emission, and PROTEOSTAT dye was detected by 559 nm diode laser excitation and 575–620 nm emission.

Confocal microscopy images of cryosections labeled by anti-1D4 or anti-4D2 antibodies or biotinylated PNA were obtained at 40× at a single z-position. High magnification images of PROTEOSTAT labeled cryosections were obtained at 100× with 3× digital zoom and a scan step of 120 nm to acquire images close to the ideal Nyquist sampling rate. Deconvolved images were generated with Huygens Essential 23.10 software (Scientific Volume Imaging, Hilversum, the Netherlands) using the standard deconvolution profile in Deconvolution Express. Maximum intensity projection images were generated, or the z-stacks were further processed in the Surface Renderer in Deconvolution Express. Quantification of TUNEL, PROTEOSTAT, and DAPI positive cells was quantified in 317 × 317 μm confocal images obtained at 40× at a single z-position that contained a segment 700–1100 μm from the optic nerve on either the superior or inferior regions of the retina. Quantification was performed in ImageJ (version 1.53n) [51], as described previously [21]. For quantification of photoreceptor cell nuclei co-labeled with TUNEL and PROTESOTAT, confocal images were obtained at 100× at a single z-position. 127 × 127 μm images containing a segment 700–1100 μm from the optic nerve on the superior region of the retina were analyzed using the Coloc 2 plugin in Fiji (version 2.1.0/1.53c). ROI (region of interest) were manually assigned for nuclei exhibiting fluorescence from TUNEL and/or PROTEOSTAT and Costes threshold regression was used to compute the Pearson’s correlation coefficient, where a positive value indicated co-labeling of a nuclei.

### Statistics

All statistical analyses were conducted using Prism 10 (GraphPad Software, San Diego, CA). No sample size calculation was performed a priori, and sample sizes used were based on previous studies. The number of images or animals analyzed (*n*) are indicated in the figure legend. Multiple comparisons were conducted using 1-way or 2-way ANOVA followed by post-hoc analysis to assess statistical significance (*P* < 0.05) of differences for individual comparisons. A two-tailed t-test was used to assess statistical significance (*P* < 0.05) between two groups. In most instances, mean values are plotted with the standard deviation or standard error, as indicated in the figure legend. For FRET data, fitted values of *E*_max_ are plotted with the standard error from the fits. The data were not assessed for normality. Qualitative experiments were conducted on at least 3 different samples to assess reproducibility.

## Supplementary information

Uncropped Western Blot

Supplementary Information

## Data Availability

All data supporting the findings of this study are available within the paper and in supplementary information files. Raw data are available from the corresponding author upon reasonable request.

## References

[1] Park PS. Constitutively active rhodopsin and retinal disease. Adv Pharm. 2014;70:1–36. 10.1016/B978-0-12-417197-8.00001-8 PMC4120657 24931191

[2] Meng D, Ragi SD, Tsang SH. Therapy in rhodopsin-mediated autosomal dominant retinitis pigmentosa. Mol Ther. 2020;28:2139–49. 32882181 10.1016/j.ymthe.2020.08.012 PMC7545001

[3] Hartong DT, Berson EL, Dryja TP. Retinitis pigmentosa. Lancet. 2006;368:1795–809. 17113430 10.1016/S0140-6736(06)69740-7

[4] Athanasiou D, Aguila M, Bellingham J, Li W, McCulley C, Reeves PJ, et al. The molecular and cellular basis of rhodopsin retinitis pigmentosa reveals potential strategies for therapy. Prog Retin Eye Res. 2018;62:1–23. 29042326 10.1016/j.preteyeres.2017.10.002 PMC5779616

[5] Ulloa-Aguirre A, Zarinan T, Gutierrez-Sagal R, Tao YX. Targeting trafficking as a therapeutic avenue for misfolded GPCRs leading to endocrine diseases. Front Endocrinol. 2022;13:934685. 10.3389/fendo.2022.934685 PMC9452723 36093106

[6] Genier S, Degrandmaison J, Lavoie CL, Gendron L, Parent JL. Monitoring the aggregation of GPCRs by fluorescence microscopy. Methods Mol Biol. 2019;1947:289–302. 30969423 10.1007/978-1-4939-9121-1_16

[7] Tao YX, Conn PM. Chaperoning G protein-coupled receptors: from cell biology to therapeutics. Endocr Rev. 2014;35:602–47. 24661201 10.1210/er.2013-1121 PMC4105357

[8] Beerepoot P, Nazari R, Salahpour A. Pharmacological chaperone approaches for rescuing GPCR mutants: current state, challenges, and screening strategies. Pharm Res. 2017;117:242–51. 10.1016/j.phrs.2016.12.036 28027910

[9] Gragg M, Park PS. Detection of misfolded rhodopsin aggregates in cells by Forster resonance energy transfer. Methods Cell Biol. 2019;149:87–105. 30616829 10.1016/bs.mcb.2018.08.007 PMC6941733

[10] Opefi CA, South K, Reynolds CA, Smith SO, Reeves PJ. Retinitis pigmentosa mutants provide insight into the role of the N-terminal cap in rhodopsin folding, structure, and function. J Biol Chem. 2013;288:33912–26. 24106275 10.1074/jbc.M113.483032 PMC3837132

[11] Kaushal S, Khorana HG. Structure and function in rhodopsin. 7. Point mutations associated with autosomal dominant retinitis pigmentosa. Biochemistry. 1994;33:6121–8. 8193125 10.1021/bi00186a011

[12] Krebs MP, Holden DC, Joshi P, Clark CL 3rd, Lee AH, Kaushal S. Molecular mechanisms of rhodopsin retinitis pigmentosa and the efficacy of pharmacological rescue. J Mol Biol. 2010;395:1063–78. 19913029 10.1016/j.jmb.2009.11.015

[13] Sung CH, Schneider BG, Agarwal N, Papermaster DS, Nathans J. Functional heterogeneity of mutant rhodopsins responsible for autosomal dominant retinitis pigmentosa. Proc Natl Acad Sci USA. 1991;88:8840–4. 1924344 10.1073/pnas.88.19.8840 PMC52606

[14] Sung CH, Davenport CM, Nathans J. Rhodopsin mutations responsible for autosomal dominant retinitis pigmentosa. Clustering of functional classes along the polypeptide chain. J Biol Chem. 1993;268:26645–9. 8253795

[15] Behnen P, Felline A, Comitato A, Di Salvo MT, Raimondi F, Gulati S, et al. A small chaperone improves folding and routing of rhodopsin mutants linked to inherited blindness. iScience. 2018;4:1–19. 30240733 10.1016/j.isci.2018.05.001 PMC6147235

[16] Liu X, Garriga P, Khorana HG. Structure and function in rhodopsin: correct folding and misfolding in two point mutants in the intradiscal domain of rhodopsin identified in retinitis pigmentosa. Proc Natl Acad Sci USA. 1996;93:4554–9. 8643442 10.1073/pnas.93.10.4554 PMC39315

[17] Hwa J, Garriga P, Liu X, Khorana HG. Structure and function in rhodopsin: packing of the helices in the transmembrane domain and folding to a tertiary structure in the intradiscal domain are coupled. Proc Natl Acad Sci USA. 1997;94:10571–6. 9380676 10.1073/pnas.94.20.10571 PMC23405

[18] Iannaccone A, Man D, Waseem N, Jennings BJ, Ganapathiraju M, Gallaher K, et al. Retinitis pigmentosa associated with rhodopsin mutations: correlation between phenotypic variability and molecular effects. Vis Res. 2006;46:4556–67. 17014888 10.1016/j.visres.2006.08.018

[19] Gragg M, Park PS. Misfolded rhodopsin mutants display variable aggregation properties. Biochim Biophys Acta. 2018;1864:2938–48. 10.1016/j.bbadis.2018.06.004 PMC6066411 29890221

[20] Dryja TP, McGee TL, Reichel E, Hahn LB, Cowley GS, Yandell DW, et al. A point mutation of the rhodopsin gene in one form of retinitis pigmentosa. Nature. 1990;343:364–6. 2137202 10.1038/343364a0

[21] Vasudevan S, Senapati S, Pendergast M, Park PS. Aggregation of rhodopsin mutants in mouse models of autosomal dominant retinitis pigmentosa. Nat Commun. 2024;15:1451. 38365903 10.1038/s41467-024-45748-4 PMC10873427

[22] Farrar GJ, Kenna P, Redmond R, Shiels D, McWilliam P, Humphries MM, et al. Autosomal dominant retinitis pigmentosa: a mutation in codon 178 of the rhodopsin gene in two families of Celtic origin. Genomics. 1991;11:1170–1. 1783387

[23] Sung CH, Davenport CM, Hennessey JC, Maumenee IH, Jacobson SG, Heckenlively JR, et al. Rhodopsin mutations in autosomal dominant retinitis pigmentosa. Proc Natl Acad Sci USA. 1991;88:6481–5. 1862076 10.1073/pnas.88.15.6481 PMC52109

[24] Bell C, Converse CA, Hammer HM, Osborne A, Haites NE. Rhodopsin mutations in a Scottish retinitis pigmentosa population, including a novel splice site mutation in intron four. Br J Ophthalmol. 1994;78:933–8. 7819178 10.1136/bjo.78.12.933 PMC504996

[25] Kim KJ, Kim C, Bok J, Kim KS, Lee EJ, Park SP, et al. Spectrum of rhodopsin mutations in Korean patients with retinitis pigmentosa. Mol Vis. 2011;17:844–53. 21677794 PMC3113629

[26] Gal A, ApfelstedtSylla E, Janecke AR, Zrenner E. Rhodopsin mutations in inherited retinal dystrophies and dysfunctions. Prog Retin Eye Res. 1997;16:51–79.

[27] Shen D, Coleman J, Chan E, Nicholson TP, Dai L, Sheppard PW, et al. Novel cell- and tissue-based assays for detecting misfolded and aggregated protein accumulation within aggresomes and inclusion bodies. Cell Biochem Biophys. 2011;60:173–85. 21132543 10.1007/s12013-010-9138-4 PMC3112480

[28] Gragg M, Kim TG, Howell S, Park PS. Wild-type opsin does not aggregate with a misfolded opsin mutant. Biochim Biophys Acta. 2016;1858:1850–9. 27117643 10.1016/j.bbamem.2016.04.013 PMC4900927

[29] Krebs MP, Collin GB, Hicks WL, Yu M, Charette JR, Shi LY, et al. Mouse models of human ocular disease for translational research. PLoS One. 2017;12:e0183837. 28859131 10.1371/journal.pone.0183837 PMC5578669

[30] Carter-Dawson L, Alvarez RA, Fong SL, Liou GI, Sperling HG, Bridges CD. Rhodopsin, 11-cis vitamin A, and interstitial retinol-binding protein (IRBP) during retinal development in normal and rd mutant mice. Dev Biol. 1986;116:431–8. 3732615 10.1016/0012-1606(86)90144-2

[31] Breton ME, Schueller AW, Lamb TD, Pugh EN Jr. Analysis of ERG a-wave amplification and kinetics in terms of the G-protein cascade of phototransduction. Invest Ophthalmol Vis Sci. 1994;35:295–309. 8300357

[32] Hood DC, Birch DG. The A-wave of the human electroretinogram and rod receptor function. Invest Ophthalmol Vis Sci. 1990;31:2070–81. 2211004

[33] Weymouth AE, Vingrys AJ. Rodent electroretinography: methods for extraction and interpretation of rod and cone responses. Prog Retin Eye Res. 2008;27:1–44. 18042420 10.1016/j.preteyeres.2007.09.003

[34] Blanks JC, Johnson LV. Specific binding of peanut lectin to a class of retinal photoreceptor cells. A species comparison. Invest Ophthalmol Vis Sci. 1984;25:546–57. 6715128

[35] Szel A, von, Schantz M, Rohlich P, Farber DB, van Veen T. Difference in PNA label intensity between short- and middle-wavelength sensitive cones in the ground squirrel retina. Invest Ophthalmol Vis Sci. 1993;34:3641–5. 8258523

[36] Sakami S, Maeda T, Bereta G, Okano K, Golczak M, Sumaroka A, et al. Probing mechanisms of photoreceptor degeneration in a new mouse model of the common form of autosomal dominant retinitis pigmentosa due to P23H opsin mutations. J Biol Chem. 2011;286:10551–67. 21224384 10.1074/jbc.M110.209759 PMC3060508

[37] Price BA, Sandoval IM, Chan F, Simons DL, Wu SM, Wensel TG, et al. Mislocalization and degradation of human P23H-rhodopsin-GFP in a knockin mouse model of retinitis pigmentosa. Invest Ophthalmol Vis Sci. 2011;52:9728–36. 22110080 10.1167/iovs.11-8654 PMC3341127

[38] Hicks D, Molday RS. Differential immunogold-dextran labeling of bovine and frog rod and cone cells using monoclonal antibodies against bovine rhodopsin. Exp Eye Res. 1986;42:55–71. 2420630 10.1016/0014-4835(86)90017-5

[39] Molday RS, MacKenzie D. Monoclonal antibodies to rhodopsin: characterization, cross-reactivity, and application as structural probes. Biochemistry. 1983;22:653–60. 6188482 10.1021/bi00272a020

[40] Vasudevan S, Park PS. Differential aggregation properties of mutant human and bovine rhodopsin. Biochemistry. 2021;60:6–18. 33356167 10.1021/acs.biochem.0c00733 PMC7863732

[41] Robichaux MA, Nguyen V, Chan F, Kailasam L, He F, Wilson JH, et al. Subcellular localization of mutant P23H rhodopsin in an RFP fusion knock-in mouse model of retinitis pigmentosa. Dis Model Mech. 2022;15:dmm049336. 10.1242/dmm.049336 PMC9092655 35275162

[42] Tam BM, Moritz OL. Dark rearing rescues P23H rhodopsin-induced retinal degeneration in a transgenic Xenopus laevis model of retinitis pigmentosa: a chromophore-dependent mechanism characterized by production of N-terminally truncated mutant rhodopsin. J Neurosci. 2007;27:9043–53. 17715341 10.1523/JNEUROSCI.2245-07.2007 PMC6672211

[43] Li D, Liu C. Conformational strains of pathogenic amyloid proteins in neurodegenerative diseases. Nat Rev Neurosci. 2022;23:523–34. 35637417 10.1038/s41583-022-00603-7

[44] Chang B, Hurd R, Wang J, Nishina P. Survey of common eye diseases in laboratory mouse strains. Invest Ophthalmol Vis Sci. 2013;54:4974–81. 23800770 10.1167/iovs.13-12289 PMC3723375

[45] Rashid K, Dannhausen K, Langmann T. Testing for known retinal degeneration mutants in mouse strains. Methods Mol Biol. 2019;1834:45–58. 30324435 10.1007/978-1-4939-8669-9_3

[46] Miller LM, Gragg M, Kim TG, Park PS. Misfolded opsin mutants display elevated beta-sheet structure. FEBS Lett. 2015;589:3119–25. 26358292 10.1016/j.febslet.2015.08.042 PMC4641566

[47] Schindelin J, Arganda-Carreras I, Frise E, Kaynig V, Longair M, Pietzsch T, et al. Fiji: an open-source platform for biological-image analysis. Nat Methods. 2012;9:676–82. 22743772 10.1038/nmeth.2019 PMC3855844

[48] Senapati S, Gragg M, Samuels IS, Parmar VM, Maeda A, Park PS. Effect of dietary docosahexaenoic acid on rhodopsin content and packing in photoreceptor cell membranes. Biochim Biophys Acta. 2018;1860:1403–13. 10.1016/j.bbamem.2018.03.030 PMC5912654 29626443

[49] Colozo AT, Vasudevan S, Park PS. Retinal degeneration in mice expressing the constitutively active G90D rhodopsin mutant. Hum Mol Genet. 2020;29:881–91. 31960909 10.1093/hmg/ddaa008 PMC7158057

[50] Vasudevan S, Samuels IS, Park PS. Gpr75 knockout mice display age-dependent cone photoreceptor cell loss. J Neurochem. 2023;167:538–55. 37840219 10.1111/jnc.15979 PMC10777681

[51] Schneider CA, Rasband WS, Eliceiri KW. NIH Image to ImageJ: 25 years of image analysis. Nat Methods. 2012;9:671–5. 22930834 10.1038/nmeth.2089 PMC5554542

