# Correction: A Y178C rhodopsin mutation causes aggregation and comparatively severe retinal degeneration

**DOI:** 10.1038/s41420-025-02346-7

**Published:** 2025-03-11

**Authors:** Sreelakshmi Vasudevan, Paul S.–H. Park

**Affiliations:** Department of Ophthalmology and Visual Sciences, Case Western Reserve University, Cleveland, OH USA

**Keywords:** Retina, Mechanisms of disease, Retinal diseases

Correction to: *Cell Death Discovery* 10.1038/s41420-025-02311-4 published online 29 January 2025

In this article a wrong version of fig. 2 has been processed during the production process.
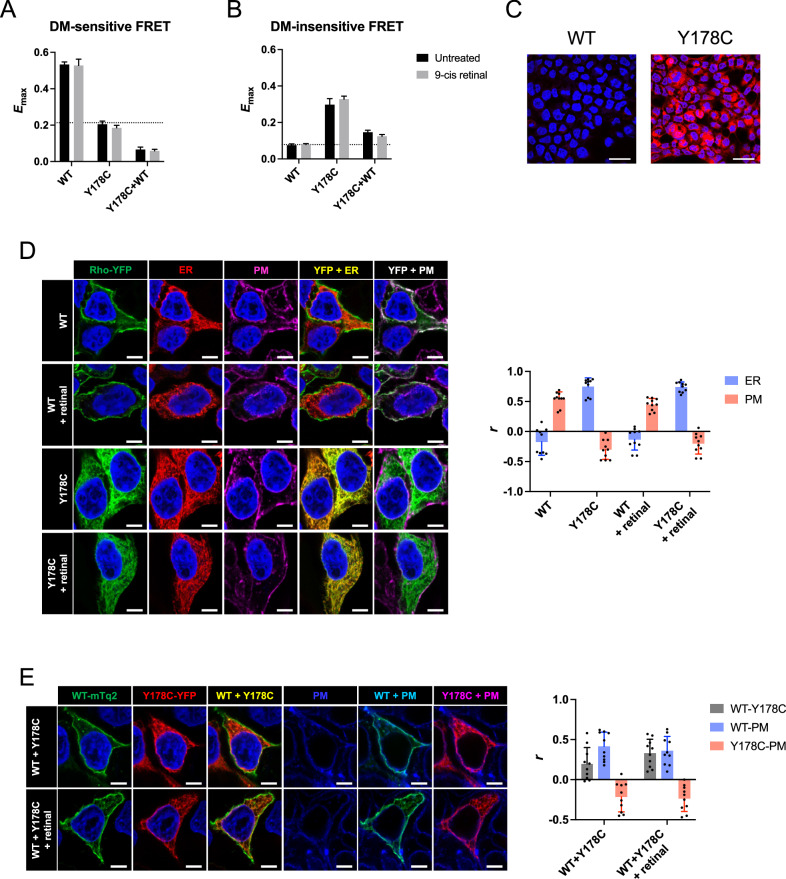


The original article has been corrected.

