# Supplementary figures and images for: A Y178C rhodopsin mutation causes aggregation and comparatively severe retinal degeneration
Source: Cell Death Discov. 2025 Jan 29;11:32. doi: 10.1038/s41420-025-02311-4 (PMC11775123; doi:10.1038/s41420-025-02311-4)

## Uncropped gel - Figure 6B

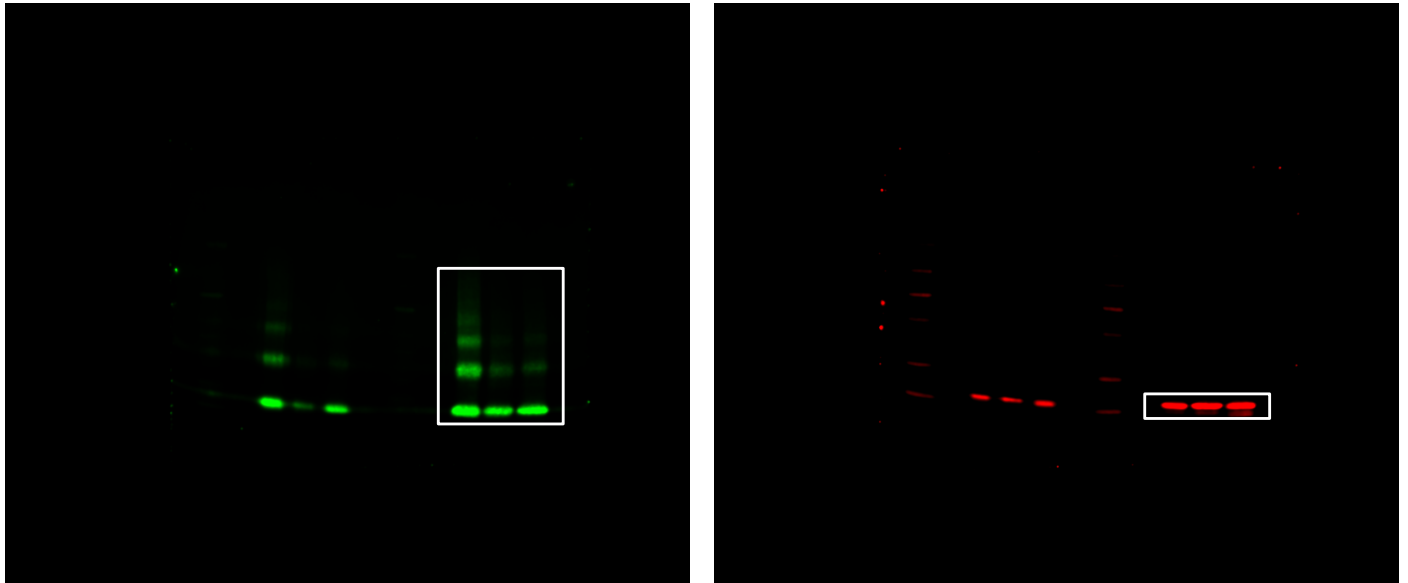

Anti-1D4 (green) and anti-GAPDH (red). Portion of blot used in figure is boxed out.

Supplement: Supplementary file 1 — Uncropped Western Blot [file 41420_2025_2311_MOESM1_ESM.pdf]
