# Supplementary material for: A Y178C rhodopsin mutation causes aggregation and comparatively severe retinal degeneration
Source: Cell Death Discov. 2025 Jan 29;11:32. doi: 10.1038/s41420-025-02311-4 (PMC11775123; doi:10.1038/s41420-025-02311-4)
Supplement: Supplementary file 2 — Supplementary Information [file 41420_2025_2311_MOESM2_ESM.pdf]

Supplementary Information

A Y178C rhodopsin mutation causes aggregation and comparatively severe retinal degeneration

Sreelakshmi Vasudevan and Paul S.–H. Park

Department of Ophthalmology and Visual Sciences, Case Western Reserve University, Cleveland, OH 44106

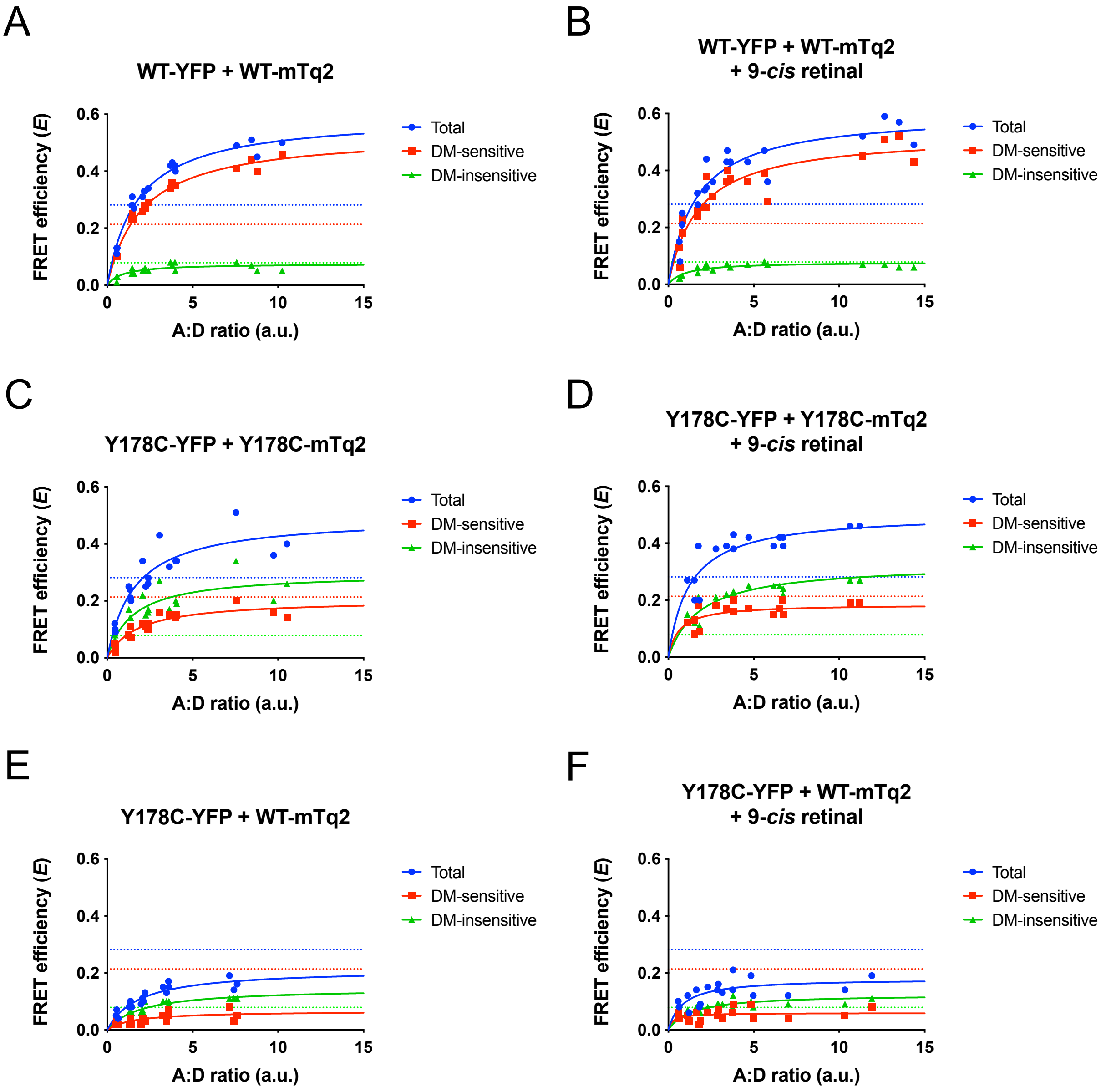

Figure 1. FRET curves. FRET curves were generated from cells expressing the indicated YFP-tagged and mTq2-tagged murine rhodopsin. Cells were either untreated or treated with 9-*cis* retinal. Total (blue), DM-sensitive (red), and DM-insensitive (green) FRET curves are shown. Each curve contains data from 4 separate experiments, which were simultaneously fit with a rectangular hyperbolic function. Fitted lines are shown and values obtained from fits are reported in Figs. 2A-2B and Supplementary Table 1. The non-specific E_{max}, defined previously [1], is indicated by the dashed lines.

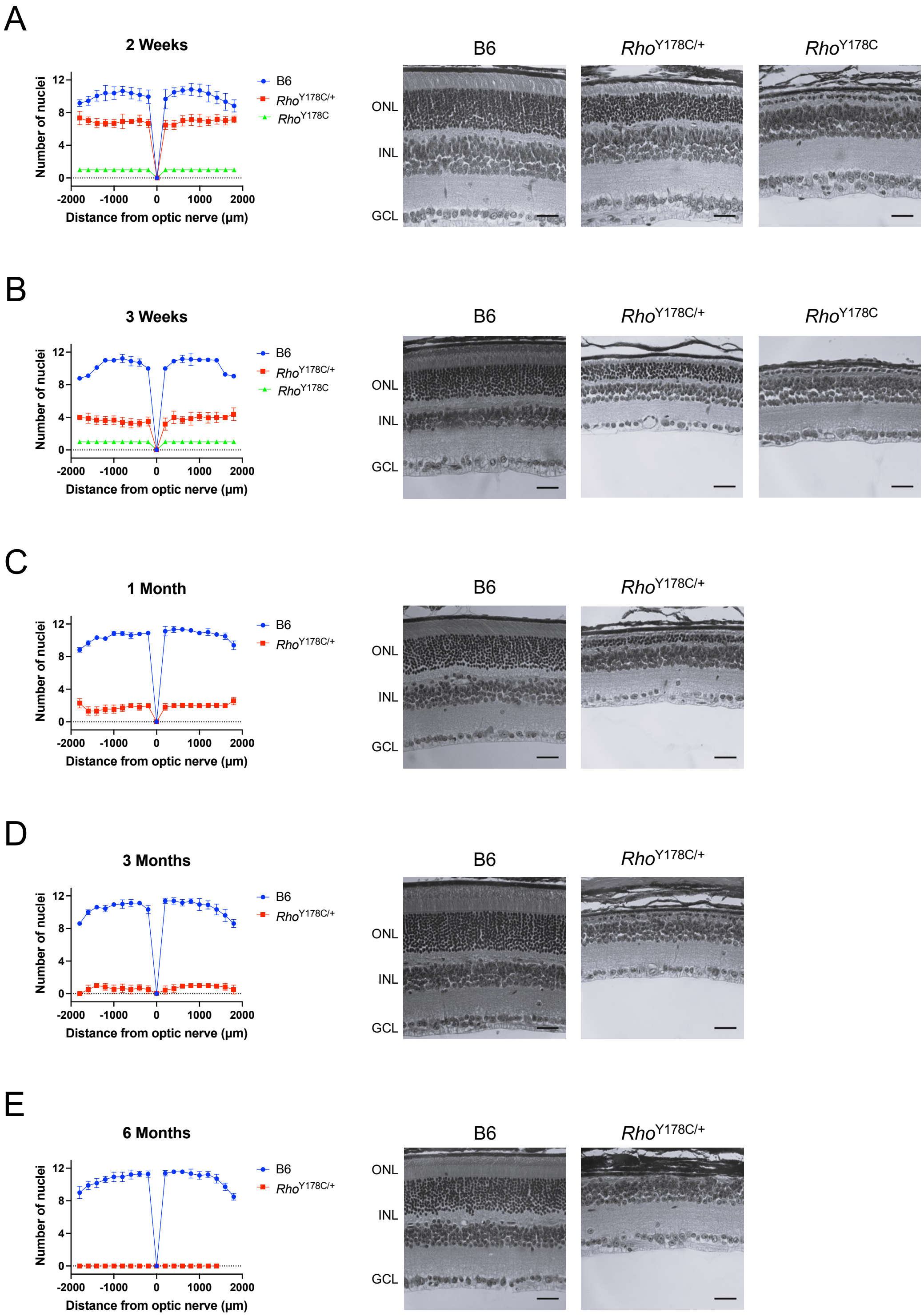

Figure 2. Photoreceptor cell loss in mice expressing the Y178C rhodopsin mutant. Uncropped images of retinal sections from B6, *Rho*$^{Y178C/+}$ and *Rho*^{Y178C} mice at different ages shown in Figs. 4A-4E are shown along with spider plots of number of photoreceptor cell nuclei. Data for B6 mice, besides that for 3-week-old mice, are those reported previously [2]. Scale bar, 50 μm.

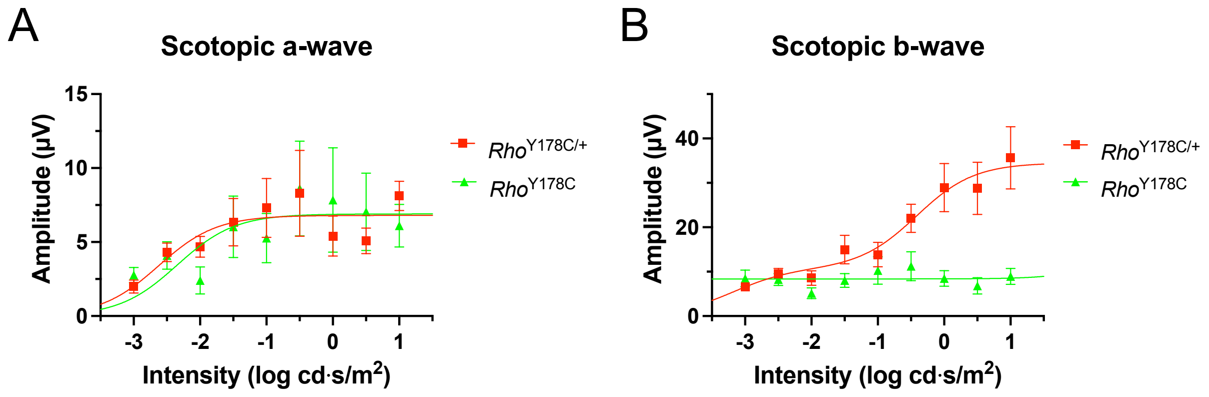

Figure 3. Scotopic a-wave and b-wave plots. Data from *Rho*$^{Y178C/+}$ and *Rho*^{Y178C} mice presented in Figs. 4H and 4I are shown on a magnified scale.

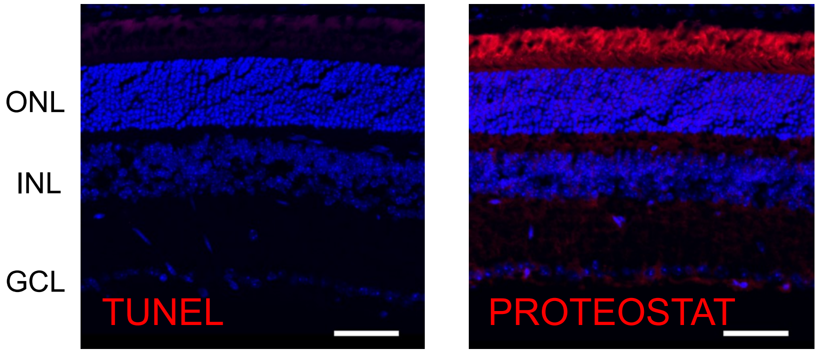

Figure 4. TUNEL and PROTEOSTAT labeling in retinal sections from B6 mice. Retinal cryosections from 2-week-old B6 mice were labeled with either TUNEL (left, red) or PROTEOSTAT (right, red). Nuclei were labeled by DAPI (blue). Scale bar, 50 µm.

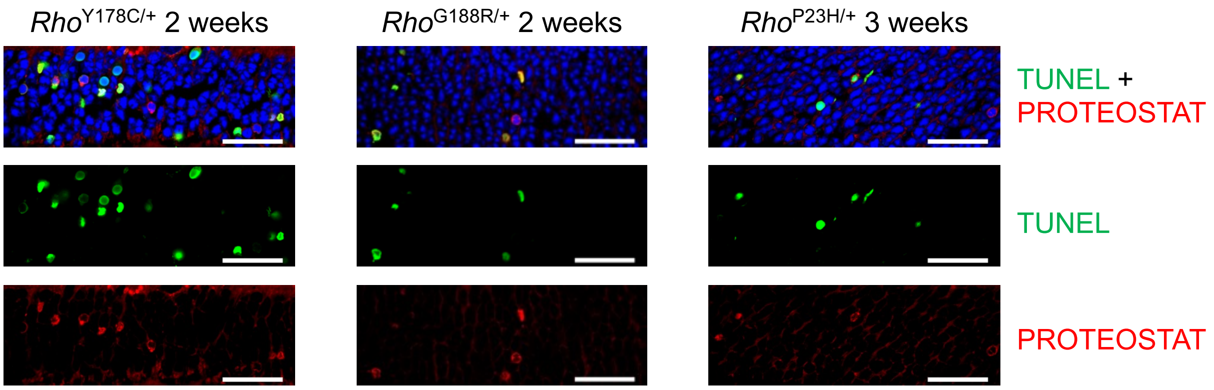

Figure 5. TUNEL and PROTEOSTAT co-labeled retinal sections. Retinal cryosections from 2-week-old *Rho*$^{Y178C/+}$ mice, 2-week-old *Rho*$^{G188R/+}$ mice, and 3-week-old *Rho*$^{P23H/+}$ mice were labeled with both TUNEL (green) and PROTEOSTAT (red). Nuclei were labeled by NucBlue (blue). Scale bar, 25 μm.

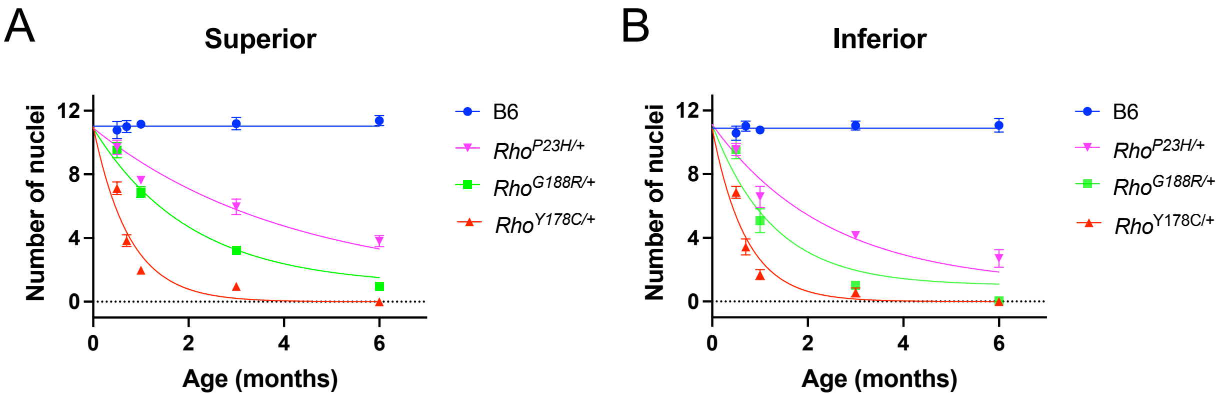

Figure 6. Kinetics of photoreceptor cell loss in B6, *Rho*$^{P23H/+}$, *Rho*$^{G188R/+}$, and *Rho*$^{Y178C/+}$ mice. Kinetic data for B6 and *Rho*$^{Y178C/+}$ mice (from Fig. 4G) are combined with data for *Rho*$^{P23H/+}$ and *Rho*$^{G188R/+}$ mice (published previously [2]) to visualize the differences in the rate of photoreceptor cell loss among the mouse lines in the superior (A) and inferior (B) retina.

Table 1. FRET curve analysis

Coexpressed receptors and treatment	Parameter					
	E_{max}			EC_{50}		
	Total	DM-sensitive	DM-insensitive	Total	DM-sensitive	DM-insensitive
**WT-YFP + WT-mTq2**	0.59 ± 0.02	0.53 ± 0.01	0.08 ± 0.01	1.76 ± 0.15	2.06 ± 0.14	0.90 ± 0.32
**WT-YFP + WT-mTq2 + 9-*cis* retinal**	0.60 ± 0.03	0.53 ± 0.04	0.08 ± 0.01	1.62 ± 0.30	1.77 ± 0.37	0.99 ± 0.27
**Y178C-YFP + Y178C-mTq2**	0.49 ± 0.04	0.20 ± 0.02	0.30 ± 0.03	1.59 ± 0.38	1.78 ± 0.42	1.49 ± 0.47
**Y178C-YFP + Y178C-mTq2 + 9-*cis* retinal**	0.50 ± 0.03	0.19 ± 0.01	0.33 ± 0.02	1.24 ± 0.28	0.63 ± 0.30	1.88 ± 0.34
**Y178C-YFP + WT-mTq2**	0.21 ± 0.02	0.07 ± 0.01	0.15 ± 0.01	1.88 ± 0.40	1.60 ± 0.85	2.05 ± 0.40
**Y178C-YFP + WT-mTq2 + 9-*cis* retinal**	0.18 ± 0.02	0.06 ± 0.01	0.12 ± 0.01	0.85 ± 0.36	0.26 ± 0.31	1.37 ± 0.38

Data in Supplementary Fig. 1 was fit to a rectangular hyperbolic function as described in the Methods. Fitted values for the maximal FRET efficiency (E_{max}) and EC_{50} are shown along with the standard errors.

Table 2. Non-specific E_{max} extra sum of squares F test analysis

**Coexpressed receptors and treatment**	***P*-value**	
	**DM-sensitive**	**DM-insensitive**
**WT-YFP + WT-mTq2**	<0.0001<br>(F (1,11) = 1441)	0.6166<br>(F (1,11) = 0.2654)
**WT-YFP + WT-mTq2<br>+ 9-*cis* retinal**	<0.0001<br>(F (1,18) = 186.9)	0.9296<br>(F (1,18) = 008021)
**Y178C-YFP + Y178C-mTq2**	0.6279<br>(F (1,17) = 0.2436)	<0.0001<br>(F (1,17) = 149.4)
**Y178C-YFP + Y178C-mTq2<br>+ 9-*cis* retinal**	0.0729<br>(F (1,16) = 3.685)	<0.0001<br>(F (1,16) = 580.2)
**Y178C-YFP + WT-mTq2**	0.0020<br>(F (1,17) = 13.33)	<0.0001<br>(F (1,17) = 79.13)
**Y178C-YFP + WT-mTq2<br>+ 9-*cis* retinal**	<0.0001<br>(F (1,17) = 34.72)	<0.0001<br>(F (1,17) = 34.72)

Data in Figs. 2A-2B and Supplementary Fig. 1 were analyzed by an extra sum of squares F test to test whether or not each E_{max} is different from the non-specific E_{max} values of 0.21 and 0.078 for DM-sensitive and DM-insensitive FRET, respectively ($P < 0.05$). P-values are reported with the F-statistic shown in parentheses.

Table 3. E_{max} comparison extra sum of squares F test analysis

Comparison groups	***P*-value**	
	**DM-sensitive**	**DM-insensitive**
**WT vs Y178C**	<0.0001<br>(F (1,35) = 89.03)	0.0006<br>(F (1,35) = 14.32)
**WT vs Y178C + WT**	0.0001<br>(F (1,35) = 18.66)	<0.0001<br>(F (1,35) = 29.18)
**WT + retinal vs Y178C + retinal**	<0.0001<br>(F (1,34) = 57.82)	<0.0001<br>(F (1,34) = 51.21)
**WT +retinal vs Y178C + WT + retinal**	0.0010<br>(F (1,35) = 12.90)	0.0001<br>(F (1,35) = 18.62)
**WT vs WT + retinal**	0.8955<br>(F (1,36) = 0.01751)	0.1673<br>(F (1,36) = 0.1673)
**Y178C vs Y178C + retinal**	0.4189<br>(F (1,33) = 0.6701)	0.4124<br>(F (1,33) = 0.6892)
**Y178C + WT vs Y178C + WT + retinal**	0.6950<br>(F (1,34) = 0.1564)	0.1788<br>(F (1,34) = 1.884)

Data in Figs. 2A-2B and Supplementary Fig. 1 were analyzed by an extra sum of squares F test to test whether or not the E_{max} values of the comparison groups indicated were significantly different ($P < 0.05$). P-values are reported with the F-statistic shown in parentheses.

Table 4. ERG analysis

Mouse	Scotopic a-wave		Scotopic b-wave				Photopic b-wave	
	R_{max} (μV)	log K_A	R_{max} (μV)	log K_A	log K_B	*f*	R_{max} (μV)	log K_A
**B6**	107 ± 3	-0.96 ± 0.07	237 ± 16	-3.17 ± 0.37	-0.63 ± 0.38	0.53 ± 0.09	60 ± 2	0.43 ± 0.07
Rho$^{Y178C/+}$	7 ± 1	-2.62 ± 0.28	35 ± 4	-3.19 ± 0.83	-0.41 ± 0.36	0.31 ± 0.10	38 ± 3	0.50 ± 0.11
Rho^{Y178C}	7 ± 1	-2.34 ± 0.39	N/A	N/A	N/A	N/A	N/A	N/A

Data in Figs. 4H-4J were fit to dose-response models described in the Methods. Fitted values are shown along with the standard errors.

Table 5. Two-tailed t-test analysis

	P-value	
	Rho$^{Y178C/+}$	***Rho***^{Y178C}
18s rRNA **normalization**	0.0174	< 0.0001
Gnat1 **normalization**	0.2905	<0.0001

Statistical analyses were conducted on data in Fig. 6A. Data from each mutant mouse line was compared to that from the B6 control by a two-tailed t-test.

Table 6. *P*-values for Tukey's post-hoc analysis

	TUNEL			PROTEOSTAT			TUNEL vs PROTEOSTAT		
	2 wks vs 3 wks	2 wks vs 1 mo	3 wks vs 1 mo	2 wks vs 3 wks	2 wks vs 1 mo	3 wks vs 1 mo	2 weeks	3 weeks	1 month
**Superior**	0.4118	0.0096	0.0003	0.0305	<0.0001	0.0090	0.1489	0.0184	0.2290
**Inferior**	0.1768	0.0032	<0.0001	0.4670	<0.0001	0.0002	0.2964	0.0593	0.2422

2-way ANOVA and Tukey's post-hoc analysis was conducted on data in Figs. 8B and 8C. Data from the superior retina: main effect of age on the number of positive cells was significant, F (2, 30) = 23.72, $P < 0.0001$; main effect of label type (TUNEL or PROTEOSTAT) on the number of positive cells was not significant, F (1, 30) = 1.673, $P = 0.2057$; interaction between age and label type was significant, F (2, 30) = 4.125, $P = 0.0261$. Data from the inferior retina: main effect of age on the number of positive cells was significant, F (2, 30) = 31.87, $P < 0.0001$; main effect of label type (TUNEL or PROTEOSTAT) on the number of positive cells was not significant, F (1, 30) = 1.458, $P = 0.237$; interaction between age and label type was not significant, F (2, 30) = 2.470, $P = 0.1016$.

Table 7. One-way ANOVA and Tukey's post-hoc analysis

	***P*-value**		
	**TUNEL only**	**PROTEOSTAT only**	**TUNEL + PROTEOSTAT**
**ANOVA**	0.0013 (F (3, 20) = 7.736)	0.0140 (F (3, 20) = 4.531)	0.0222 (F (3, 20) = 3.992)
***Rho*$^{Y178C/+}$ (2 w) vs *Rho*$^{Y178C/+}$ (3 w)**	0.9787	0.9930	0.9997
***Rho*$^{Y178C/+}$ (2 w) vs *Rho*$^{G188R/+}$ (2 w)**	0.0259	0.9117	0.0369
***Rho*$^{Y178C/+}$ (2 w) vs *Rho*$^{P23H/+}$ (3 w)**	0.0181	0.0671	0.8228
***Rho*$^{Y178C/+}$ (3 w) vs *Rho*$^{G188R/+}$ (2 w)**	0.0110	0.9794	0.0300
***Rho*$^{Y178C/+}$ (3 w) vs *Rho*$^{P23H/+}$ (3 w)**	0.0076	0.0388	0.7718
***Rho*$^{G188R/+}$ (2 w) vs *Rho*$^{P23H/+}$ (3 w)**	0.9983	0.0169	0.1933

Statistical analyses were conducted on data in Fig. 8E. *P*-values are shown for ANOVA and Tukey's post-hoc analysis for individual comparisons. The *F*-statistic is shown in parentheses for the ANOVA analysis.

References

1 Gragg, M. & Park, P. S. Misfolded rhodopsin mutants display variable aggregation properties. *Biochim. Biophys. Acta* **1864**, 2938-2948 (2018). https://doi.org/10.1016/j.bbadis.2018.06.004

2 Vasudevan, S., Senapati, S., Pendergast, M. & Park, P. S. Aggregation of rhodopsin mutants in mouse models of autosomal dominant retinitis pigmentosa. *Nat Commun* **15**, 1451 (2024). https://doi.org/10.1038/s41467-024-45748-4
